# New *Streptomyces*-Derived Antibacterial Compounds Targeting Gram-Positive Bacteria: A Systematic Review

**DOI:** 10.1155/tswj/6659874

**Published:** 2025-09-24

**Authors:** Soumia Ait Assou, Mohammed El Hassouni

**Affiliations:** Department of Biology, Biotechnology, Environment, Agri-Food and Health Laboratory, Faculty of Sciences Dhar El Mahraz, Sidi Mohamed Ben Abdellah University, Fez, Morocco

**Keywords:** antibacterial activity, Gram-positive bacteria, natural products, *Streptomyces*

## Abstract

Bacterial infections, particularly those caused by Gram-positive bacteria like vancomycin-resistant enterococci (VRE) and methicillin-resistant *Staphylococcus aureus* (MRSA), are a growing concern. This review highlights the potential of the *Streptomyces* genus in producing novel antibacterial compounds against Gram-positive bacteria. The study was carried out following the guidelines of the Preferred Reporting Items for Systematic Reviews and Meta-Analysis (PRISMA). To gather relevant literature on novel anti-Gram-positive bacteria compounds produced by *Streptomyces*, a web search was conducted using several databases, including Scopus, PubMed, ScienceDirect, and Google Scholar, covering the period from January 2013 to mid-2024. The search terms employed in this study included “*Streptomyces*,” “antimicrobial/antibacterial activity,” “compounds,” and “Gram-positive bacteria.” Consequently, a total of 248 *Streptomyces*-derived compounds were featured across the 96 eligible studies. These compounds include 100 polyketides (58 aromatic polyketides, 30 macrolides, and 12 other polyketides), 72 peptides (67 nonribosomal peptides [52 typical cyclic peptides and 15 lipopeptides] and 5 ribosomal peptides), 23 terpenoids, five polyketides–terpenoids, six alkaloids, 12 phenazines, 11 nucleoside antibiotics, and 19 other compounds belonging to distinct chemical classes. The results emphasize that *Streptomyces* is an unlimited source of naturally occurring compounds with various structural variations that can occasionally have targeted action against a range of pathogenic Gram-positive bacteria.

## 1. Introduction

One of the most significant public health issues that has to be addressed globally is antimicrobial resistance (AMR) [1, 2]. According to a 2016 UK government report, AMR may kill 10 million people annually by 2050 [3]. Multidrug-resistant microbial strains, some resistant to first-line antibiotics, are becoming more prevalent as antibiotic efficacy continues to decline [4].

Multidrug resistance in Gram-negative bacteria has received a lot of attention worldwide, but Gram-positive bacteria are also a major concern [5]. The World Health Organization (WHO) recently published a global priority pathogen list and categorized them as critical, high, and medium antibiotic-resistant bacteria based on the urgent need for new antibiotics [6]. Multidrug-resistant bacteria like vancomycin-resistant *Enterococcus faecium* (VREfm) and MRSA stand out among the most common Gram-positive bacteria that can cause serious infections and are regarded as a major problem and health hazard [6]. Because of the emergence of these multidrug-resistant bacteria, it is crucial to increase research and development efforts to discover and develop new antibiotics as well as look into alternative treatment options [7].

Since Fleming's unintentional discovery of penicillin in 1928, microorganisms have provided a never-ending source of bioactive compounds with a wide range of biological functions, most notably antimicrobial activity. In fact, bacteria are the primary source of the majority of antibiotics available on the market. With around 12,000 known bioactive metabolites, actinomycetes are among the most abundant bacteria for producing bioactive compounds, including antibiotics. Approximately 80% of these metabolites are produced by the *Streptomyces* genus, making it the greatest producer [8].

Numerous biosynthetic gene clusters (BGCs) are closely linked to the production of secondary metabolites by actinomycetes, especially *Streptomyces*, according to genomic investigations of these bacteria. According to Nett et al. [9], between half and three-quarters of these BGCs encode enzyme complexes, such as nonribosomal peptide synthetases (NRPSs) and polyketide synthases (PKSs), and their hybrid compounds. It is interesting to note that despite having a similar biosynthesis process, polyketides generated by various actinomycete strains exhibit notable chemodiversity [10]. Numerous biological actions exhibited by these metabolites have been established for the synthesis of various medications, primarily antibiotics [11].

This review aims to identify antibacterial compounds active specifically against Gram-positive bacteria, derived from the *Streptomyces* genus, discovered between 2013 and mid-2024. It emphasizes the isolation source of the *Streptomyces* species/strains, the chemical classes of bioactive compounds, and their antibacterial activity, as well as the most potent compounds and their chemical structures.

Some studies have specifically reviewed Gram-positive bacteria inhibited by compounds derived from marine *Actinobacteria*, such as anti-*Listeria* compounds [12] or the work of Kemung et al. [13] on potential drug candidates against MRSA derived from the *Streptomyces* strains with a focus on those from only underexplored biotopes. Additionally, several studies have focused on specific classes of novel compounds from marine actinomycetes or the *Streptomyces* genus [14, 15]. Some studies focused on compounds from *Streptomyces* with various biological activities, specifically over the course of 1 year. For example, Lacey and Rutledge [16] conducted a review on new *Streptomyces* compounds, reviewing studies published in 2020 that investigated a range of biological activities.

Our study stands out with a distinctive approach. It consolidates information exclusively on new anti-Gram-positive bacteria compounds derived from *Streptomyces* species or strains isolated from diverse environments and biotopes. By focusing on compounds identified between January 2013 and mid-2024, this review offers a clear classification of these compounds. Our aim is to provide the scientific community with a solid knowledge base, facilitating future research efforts and enabling the identification of promising compounds with significant therapeutic potential.

## 2. Methods

### 2.1. Literature Search Strategy

This review was performed according to the PRISMA checklist. We carried out a thorough and systematic search across multiple databases to identify all relevant studies on novel antibacterial compounds against Gram-positive bacteria, produced by the *Streptomyces* genus, over the past decade (from January 2013 to June 2024). A deep search was conducted via the databases PubMed (http://www.ncbi.nlm.nih.gov/pubmed), Google Scholar (http://scholar.google.com), Scopus (http://www.scopus.com), and online collection ScienceDirect (http://www.sciencedirect.com). Articles in the English language were searched using different phrases in combination using Boolean operators (“OR”/“AND”) such as “*Streptomyces*,” “antimicrobial/antibacterial activity,” “compounds,” and “Gram-positive bacteria.” The search methodology was illustrated following PRISMA 2020 method guidelines along with the studies included or excluded, explaining the reasons for exclusion.

### 2.2. Criteria for Inclusion and Exclusion of Studies

To obtain representative information, inclusion and exclusion criteria were applied when searching for scientific articles.

➢ Inclusion criteria
  i. Compounds from *Streptomyces* spp. that were tested for antibacterial activity.
  ii. Anti-Gram-positive bacteria activity was from *Streptomyces* spp. compounds.
  iii. Antibacterial activity reported in MIC value.
➢ Exclusion criteria
  i. Not a compound produced by *Streptomyces* sp.
  ii. Using different techniques apart from the minimum inhibitory concentration (MIC) technique: 50% inhibitory concentration (IC_50_), 50% minimum inhibitory concentration (MIC_50_), 90% minimum inhibitory concentration (MIC_90_), and the diameter of inhibition zone (DIZ).
  iii. Broad spectrum of activity (anti-Gram-negative bacteria and or anti-fungi).

### 2.3. Selection Process

The authors conducted an independent review of titles and abstracts to evaluate their relevance based on defined inclusion criteria. Full texts of studies that satisfied these criteria were then obtained for a more thorough assessment. Both authors engaged in this full-text screening, collaboratively resolving any disagreements through discussion and consensus. Following this screening, a final selection of studies was chosen for inclusion in the review, ensuring that all met the eligibility criteria and aligned with the review's objectives. The initial screening and data extraction were led by the first author, S.A.A., while the second author, M.E.H., provided guidance and oversight throughout the process. Ultimately, both authors participated in the final decision-making to address any discrepancies in the study selection.

### 2.4. Data Collection Process

The data was collected by the first author (S.A.A.). To ensure accuracy and consistency, all extracted data underwent a thorough double-checking. Any discrepancies were addressed through discussions between the two authors. The variables extracted included the publication year, *Streptomyces* species or strain, isolation source (such as marine sediment, soil, and endophyte), the Gram-positive bacteria tested, the chemical class of each bioactive compound and its MIC value, and the structures of the most potent compounds. When MIC values were reported in molar units, they were converted to microgram per milliliter using the molecular weight of each compound to ensure consistency across the studies.

### 2.5. Study Risk of Bias Assessment

This review focuses on the antibacterial activity of natural products from *Streptomyces* species or strains. Consequently, the majority of the studies included are experimental laboratory studies rather than clinical trials or observational studies. As a result, conventional tools for assessing methodological bias are not applicable.

### 2.6. Data Synthesis

The extracted data were synthesized qualitatively due to the heterogeneity of study designs, compound classes, and target bacterial strains involved. Antibacterial activity was grouped and summarized according to the chemical class of the compounds. To provide a clear overview, for each chemical class, a summary table was constructed to present MIC values alongside the corresponding *Streptomyces* strains or species, their isolation sources, and the targeted Gram-positive bacteria. Compounds with particularly low MIC values were highlighted, and their chemical structures were included. No statistical meta-analysis was carried out.

## 3. Results

### 3.1. Study Selection

**Figure 1:**
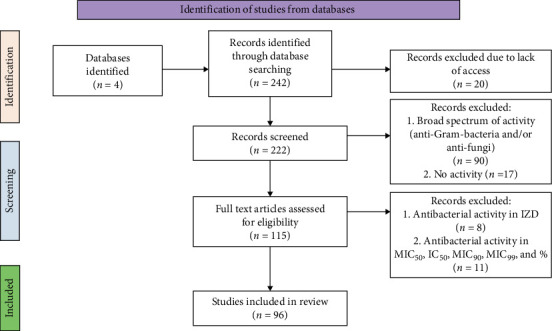
Identification of articles on inhibition of Gram^+^ bacteria by *Streptomyces* spp. compounds using the PRISMA 2020 method [17].

Figure 1 demonstrates the article selection method, PRISMA 2020 [17]. The literature search identified 242 scientific articles. A screening process was then conducted to select only those relevant to the study. In the initial screening, 107 articles were excluded because the reported compounds exhibited a broad spectrum of activity, inhibiting both Gram-negative bacteria and fungal strains, or exhibiting no activity. This step resulted in 115 articles being retained for further evaluation. During the second screening phase, only articles reporting antibacterial activity against Gram-positive bacteria with clearly defined MIC values were retained. A further 19 articles were excluded, due to inconsistent reporting formats such as IC_50_, MIC_50_, MIC_99_, or percentage-based values. Therefore, only 96 of the studies met the eligibility criteria and were considered in the final systematic review (Figure 1).

### 3.2. Study Characteristics

The included articles in this study focused on the isolation of one or more compounds derived from a single strain or species of *Streptomyces*. To assess antibacterial activity, all studies employed *in vitro* laboratory methods, particularly the broth microdilution method, and reported MIC values in microgram per milliliter. The Gram-positive bacterial strains examined across all the articles include, in particular, strains of *Staphylococcus* (*aureus*, *saprophyticus*, *epidermidis*, *haemolyticus*, *warneri*, and *simulans*), *Bacillus* (*subtilis*, *cereus*, *anthracis*, *thuringiensis*, and *megaterium*), *Enterococcus* (*faecalis*, *faecium*, *hirae*, and *gallinarum*), *Micrococcus luteus*, *Listeria* (*monocytogenes* and *ivanovii* subsp. *ivanovii*), *Mycobacterium* (*tuberculosis*, *smegmatis*, *intracellulare*, *bovis*, *avium*, *abscessus*, and *aurum*), *Streptococcus* (*agalactiae*, *pneumoniae*, *pyogenes*, and *anginosus*), *Lactobacillus* (*brevis*, *bulgaricus*, and *sakei* subsp. *sakei*), *Clostridium* (*difficile*, *bifermentans*, *butyricum*, *indolis*, *innocuum*, *limosum*, *perfringens*, and *ramosum*), and *Kocuria rhizophila*.

### 3.3. Source of Isolation of *Streptomyces* Species/Strains

**Figure 2:**
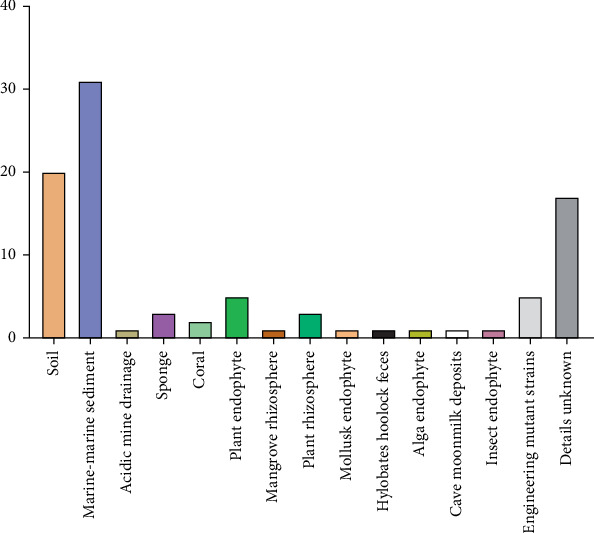
Sources of *Streptomyces* species/strains.

In reviewing the 96 publications, it was found that 93 studies reported on a distinctive species or strain, while the remaining three studies focused, each one, on a single strain that was also among the 93 distinctive strains. As a result, 93 strains were documented, originating from different environmental sources.  Figure 2 illustrates this diversity, showing that the isolation sources range from terrestrial environments (soil, acidic mine drainage, plants, insects, and animals) to marine environments (marine sediments, sponges, and corals). Marine sediments alone account for 33.33% (*n* = 31) of the described *Streptomyces* strains, followed by terrestrial soil with 21.50% (*n* = 20). Notably, a significant proportion of the publications (18.27%, *n* = 17) did not specify the environmental source of the *Streptomyces* strain (Figure 2).

### 3.4. Classes of Anti-Gram-Positive Bacteria Compounds Isolated From the Genus *Streptomyces* in the Past Decade

The 96 articles included in the present review were further analyzed, leading to the identification of 248 compounds classified into several classes.

#### 3.4.1. Polyketides

Polyketides constitute a diverse class of natural metabolites characterized by multiple *β*-hydroxyketone or *β*-hydroxyaldehyde groups. These compounds are synthesized by enzymatic complexes known as PKSs.

In *Streptomyces*, three main types of PKSs (Type I, Type II, and Type III) are responsible for polyketide biosynthesis. Type I PKSs specifically produce macrolides, while Type II PKSs are involved in the synthesis of aromatic polyketides. Based on their polyphenolic cyclic systems and biosynthetic pathways, aromatic polyketides are further classified into three major groups: anthracyclines, angucyclines, and tetracyclines, alongside several other diverse subclasses [18, 19].

**Figure 3:**
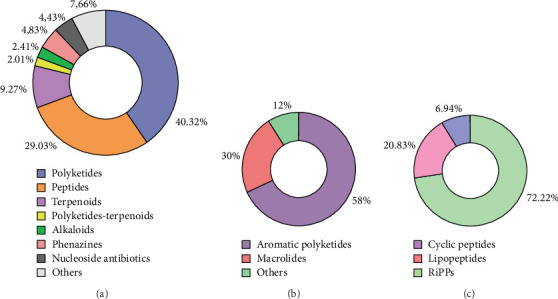
Main classes of compounds isolated from the *Streptomyces* genus between 2013 and mid-2024. (a) All compound classes, (b) subclass of polyketides, and (c) subclass of peptides.

As illustrated in Figure 3, polyketides represent the most isolated class of anti-Gram-positive bacterial compounds from the *Streptomyces* genus since 2013, with 100 compounds identified, accounting for 40.32% of the total compounds (Figure 3a). These polyketides are subdivided into:

• Aromatic polyketides: representing the largest subclass, with 58 compounds (58%), primarily including angucyclines, anthracyclines, and xanthones.
• Macrolides: the second-largest subclass, comprising 30 molecules (30%).
• Other polyketides: a diverse group of 12 compounds (12%) belonging to various other polyketide families (Figure 3b).

#### 3.4.2. Peptides

Microbial peptides with antimicrobial activity are categorized into two main classes: nonribosomal peptides (NRPs) and ribosomal peptides.

• NRPs: These are structurally diverse secondary metabolites produced by fungi and bacteria through multimodular enzymes called NRPSs, independently of ribosomes [20]. NRPs principally include cyclic peptides (typically composed of two to eight amino acids) and lipopeptides. These peptides have either linear or cyclic structures, characterized by an N-terminus hydrophobic fatty acid tail [21].
• Ribosomal peptides: These peptides are ribosomally synthesized and posttranslationally modified peptide natural products (RiPPs). They mainly include lassopeptides, thiopeptides, and lanthipeptides. Lassopeptides have a macrocyclic ring formed by seven to nine N-terminal amino acid residues that trap the C-terminus [22]. Thiopeptides (or thiazolyl peptides) are sulfur-rich peptides [23]. Lanthipeptides are distinguished by thioether-bridged amino acids, such as lanthionine (Lan) and methyllanthionine (MeLan) [24].

Since 2013, 72 peptide compounds (29.03%) derived from *Streptomyces* with specific activity against Gram-positive bacteria have been identified (Figure 3):

• NRPs: with 67 (52 cyclic peptides [72.22%] and 15 lipopeptides [20.83%]).
• RiPPs: with five (6.94%), represented by lassopeptides, thiopeptides, and lanthipeptides (Figure 3c).

#### 3.4.3. Terpenoids

Terpenoids are natural chemical compounds synthesized through the condensation of isopentenyl diphosphate (IPP) and its isomer, dimethylallyl diphosphate (DMAPP). Meroterpenoids and diterpenoids mainly represent them. Among the meroterpenoids, napyradiomycins form a prominent subclass. While numerous terpenoid metabolites have been isolated from plants and fungi, bacterial terpenoids remain relatively rare [25]. Except for geosmin, a terpene responsible for the characteristic earthy odor of *Streptomyces*, these bacteria are considered a limited source of terpenes [26]. As shown in Figure 3a, terpenoids represent the third-largest class (23 compounds [9.27%]) of compounds exhibiting specific activity against Gram-positive bacteria, isolated from the *Streptomyces* genus since 2013.

#### 3.4.4. Polyketides–Terpenoids

Polyketides–terpenoids are hybrid natural products derived from both polyketide and terpenoid biosynthetic pathways. Over the past decade, five (2.01%) polyketides–terpenoids with antibacterial activity against Gram-positive bacteria have been identified (Figure 3a).

#### 3.4.5. Alkaloids

Alkaloids are the main secondary metabolites of actinomycetes and one of the most medicinal types of compounds. Most of these nitrogen-containing molecules possess complex ring structures with important pharmacological activity [27]. In this review, six alkaloids (2.41%), targeting Gram-positive bacteria, were isolated from the *Streptomyces* genus over the past decade (Figure 3a).

#### 3.4.6. Phenazines

Phenazines are a large family of nitrogen-containing natural compounds characterized by two benzene rings linked via two nitrogen atoms. They exhibit a wide range of biological activities, including antibacterial, antitumor, and antiparasitic properties [28]. In this review, we report the identification of 12 new phenazines (4.83%) specifically inhibiting Gram-positive bacteria, isolated from the *Streptomyces* genus over the past decade, from 2013 to mid-2024 (Figure 3a).

#### 3.4.7. Nucleoside Antibiotics

Nucleoside antibiotics are an important family of microbial natural products derived from nucleosides and nucleotides. They exhibit various biological activities, such as antibacterial, antifungal, antiviral, insecticidal, immunostimulative, and antitumor activities. Nucleoside antibiotics can be grouped into two classes, *C*-nucleosides and *N*-nucleosides, in which a sugar and a nucleobase are linked via a C–C bond or a C–N bond, respectively [29]. Over the past decade, 11 (4.43%) nucleoside antibiotics with antibacterial activity against Gram-positive bacteria have been identified (Figure 3a).

#### 3.4.8. Other Compounds

In addition to the aforementioned classes, 19 other compounds (7.66%) with activity against Gram-positive bacteria have been isolated over the past decade (Figure 3a). These compounds belong to distinct chemical classes, including dimeric cinnamoyl lipids, liposidomycin congeners, siderophores, gilvocarcin-type aryl-C-glycosides, and tetracene derivatives.

### 3.5. Anti-Gram-Positive Bacteria Activity of Newly Isolated Compounds

**Figure 4:**
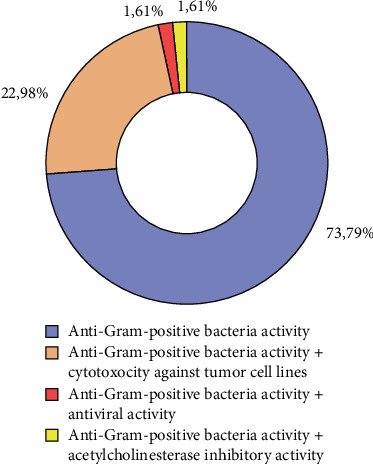
Distribution of biological activities of newly identified compounds.

The antibacterial activity of the 248 novel compounds was analyzed according to their chemical classification. It is important to note that some compounds exhibited additional biological activities, including cytotoxic activity against tumor cells (22.98%, *n* = 57), antiviral activity (1.61%, *n* = 4), and acetylcholinesterase inhibitory activity (1.61%, *n* = 4) in addition to their antibacterial effect against Gram-positive bacteria (Figure 4).

In the following section, the previously unidentified compounds with exclusive activity against Gram-positive bacteria that were first isolated from the *Streptomyces* genus, since 2013 to mid-2024, are listed according to their chemical classification, along with the description of the potent ones, their chemical structures, and their structure–activity relationships (SARs), if available.

#### 3.5.1. Polyketides

##### 3.5.1.1. Aromatic Polyketides

As shown in Table 1, there have been numerous reports of aromatic polyketides produced by the *Streptomyces* genus during the last decade, with a total of 58 compounds. These polyketides belong to different families (angucyclines, anthracyclines, xanthones, etc.) and show antibacterial activity against Gram-positive bacteria, including MRSA and VRE.

**Figure 5:**
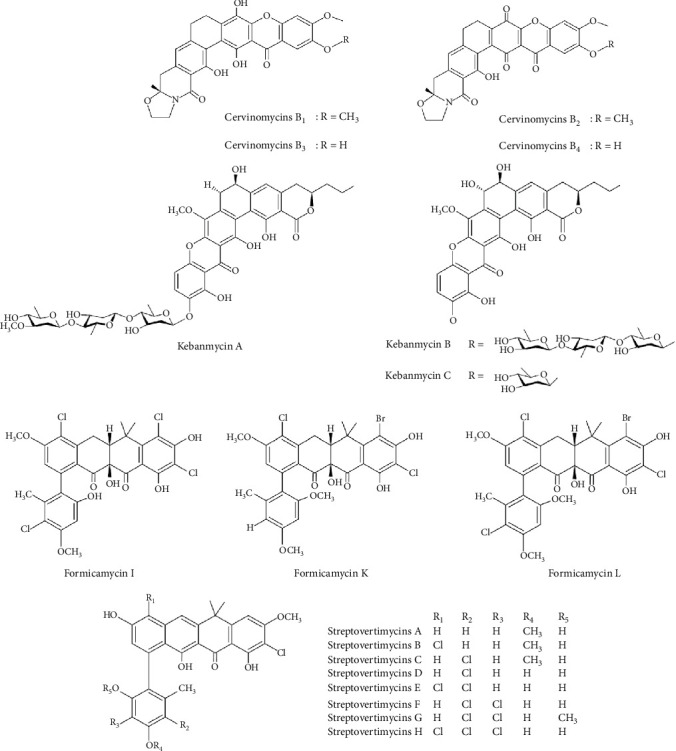
Chemical structures of aromatic polyketides from *Streptomyces* with potent antibacterial activity against Gram-positive bacteria.

Aromatic polyketide compounds with the lowest MIC values and therefore effective concentrations were derived from the terrestrial *Streptomyces* CPCC 204980. This species produced four notable polycyclic xanthones, namely, cervinomycins B_1−4_, which showed very good anti-Gram-positive bacteria activity, with MICs ranging from 0.008 to 0.5 *μ*g/mL against *S. aureus* ATCC 33591, *S. aureus* 16-30, *Enterococcus faecalis* ATCC 51299, and *E. faecium* ATCC 700221 (Table 1 and Figure 5) [30].

Another group of highly polycyclic xanthones, known as kebanmycins A–C, was isolated from a mangrove-derived actinomycete, *Streptomyces* sp. SCSIO 40068 (Figure 5). These compounds exhibited significant antibacterial activity against several strains of *S. aureus* (ATCC 29213, MRSA shhs-A1, MRSA 1862, MRSA 669, and MRSA 991) with MICs of 0.125–0.5 *μ*g/mL for kebanmycin A, 1–2 *μ*g/mL for kebanmycin B, and 0.5–4 *μ*g/mL for kebanmycin C. Notably, kebanmycin B displayed the strongest activity against *Bacillus subtilis* 1064, with an MIC of 1 *μ*g/mL, while kebanmycin A and kebanmycin C showed MICs of > 64 and 4 *μ*g/mL, respectively (Table 1) [31].

From a new *Streptomyces* species, named *S. formicae*, isolated from the African fungus-growing plant-ant *Tetraponera penzigi*, 15 novel pentacyclic polyketides were purified. Among them, formicamycins I, K, and L stand out as the most potent, displaying antibacterial activity against *B. subtilis*, MRSA, and VREfm with MIC values ranging from 0.80 to 3.26 *μ*g/mL (1.25–5 *μ*M) (Table 1 and Figure 5) [32]. To examine their SAR, the growth of *B*. *subtilis* was examined in liquid media supplemented with 0.01–100 *μ*M of isolated formicamycins. Dose–response analyses showed that all compounds effectively inhibit the growth of *B*. *subtilis*, with enhanced effectiveness noted for compounds containing an increasing number of chlorine atoms (formicamycins D–L). Notably, the brominated compounds (formicamycins K and L) appear to exhibit a slightly greater potency compared to their chlorinated counterparts [32].

Additional strongly aromatic polyketide compounds were fasamycins isolated from *S. morookaense*. This strain produced eight fasamycins compounds designated streptovertimycins A–H (Figure 5). These compounds show strong inhibitory potential at low concentrations, such as streptovertimycin G at 0.63 and 1.25 *μ*g/mL against MRSA and VREfm, respectively (Table 1) [33]. Comparative SAR analysis suggests that a free hydroxyl group at C-3 is beneficial for the activity. Furthermore, the 5-*O*-methyl bearing streptovertimycin G increases potency two- to four-fold compared to its 5-OH variant (streptovertimycin F) [33].

##### 3.5.1.2. Macrolides

Macrolides are a large family of natural products with various biological activities that have attracted interest from the pharmaceutical community [50]. Most macrolide antibiotics are typically characterized by a lactone ring and one or more sugar moieties. Over the past decade, 30 novel macrolides with antibacterial activity against Gram-positive bacteria have been identified from the *Streptomyces* genus (Table 2).

**Figure 6:**
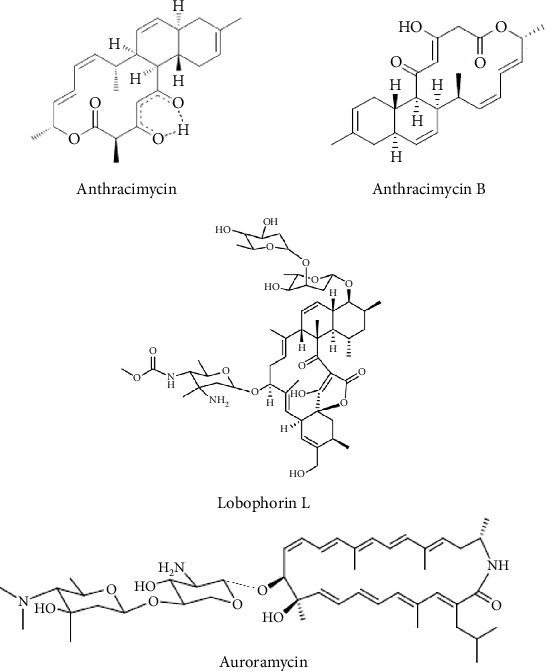
Chemical structures of macrolides from *Streptomyces* with potent antibacterial activity against Gram-positive bacteria.

One remarkable discovery is anthracimycin (Figure 6), a macrolide isolated from *Streptomyces* sp. CNH365, derived from marine sediments of the coast of Santa Barbara in the Pacific Ocean. Anthracimycin demonstrates potent inhibitory activity against Gram-positive bacteria, particularly *Bacillus anthracis* (strain UM23C1-1), with an exceptionally low minimum MIC value of 0.031 *μ*g/mL, and 0.0625 and 0.125 *μ*g/mL against *S. aureus* ATCC 13709 and *E. faecalis* ATCC 29212, respectively (Table 2) [51].

Five years later, a structurally related compound, anthracimycin B (Figure 6), was isolated from the deep-sea *S. cyaneofuscatus* M-169 through bioassay-guided fractionation. Although less potent than anthracimycin, anthracimycin B still exhibited significant antibacterial activity against tested pathogens (*S*. *aureus* MB5393 [MRSA] and ATCC 29213 [MSSA]), vancomycin-sensitive *E. faecium* CL144754 (VSE), and E. faecalis CL144492 (VSE) with MIC values ranging from 0.125 to 8 *μ*g/mL (Table 2) [52]. A preliminary SAR hypothesis suggests that the methyl group at C-2 present in anthracimycin plays an important role in its strong antibacterial activity in comparison with anthracimycin B [52].

Another noteworthy group is the spirotetronate macrolides, recognized for their complex chemical structures, diverse biological activities, and promising pharmacological potential [66]. Over the past decade, five new spirotetronate compounds active against Gram-positive bacteria have been discovered (Table 2). Among them, lobophorin L (Figure 6), isolated from the marine-derived *Streptomyces* sp. 4506, displayed strong to moderate antibacterial activity against *M. luteus* and *Bacillus thuringiensis*, with MIC values ranging from 4 to 8 *μ*g/mL (Table 2) [53]. SAR insights indicate that the degree of sugar substitution at C-9 correlates positively with antibacterial potency: The two sugar moiety substitution in lobophorin L (MIC of 8 *μ*g/mL) showed the strongest antibacterial activity compared with lobophorin M (one sugar moiety substitution, with MIC above 128 *μ*g/mL) against *M. luteus* [53]. Using the CRISPR-Cas9 gene cluster activation strategy, Lim et al. [54] reported the discovery of a novel doubly glycosylated 24-membered polyene macrolactam, named auroramycin, from a silent BGC in *S. roseosporus* NRRL 15998 (Figure 6). Auroramycin showed potent antibacterial activity against MRSA with MICs of 1–2 *μ*g/mL. It also inhibited other Gram-positive pathogenic bacteria such as *S. aureus* (vancomycin-intermediate (VI) MRSA) and vancomycin-resistant* E. faecalis* (VRE) with MICs ranging from 1 to 4 *μ*g/mL (Table 2).

##### 3.5.1.3. Other Polyketide Compounds

As shown in Table 3, 12 additional compounds belonging to different classes of polyketides have been reported within the *Streptomyces* genus over the last decade, with half of them displaying negligible inhibitory effect (MIC > 100* μ*g/mL) against Gram-positive bacteria.

#### 3.5.2. Peptide Compounds

There have been 71 natural peptide antibiotic products derived from *Streptomyces*, known for their biological activity against Gram-positive bacteria, since 2013. Fifty-two of these peptides are cyclic peptides and cyclic peptides containing piperazic acid (Table 4).

##### 3.5.2.1. Cyclic Peptides

Among the cyclic peptides identified, actinomycins emerged as the most potent cyclic peptides exhibiting significant inhibitory activity against Gram-positive bacteria. These compounds, isolated from various *Streptomyces* species, are well-known chromopeptides consisting of two cyclic pentapeptide lactones (*α*- and *β*-rings) attached through amide bonds to a central phenoxazinone chromophore [87].

**Figure 7:**
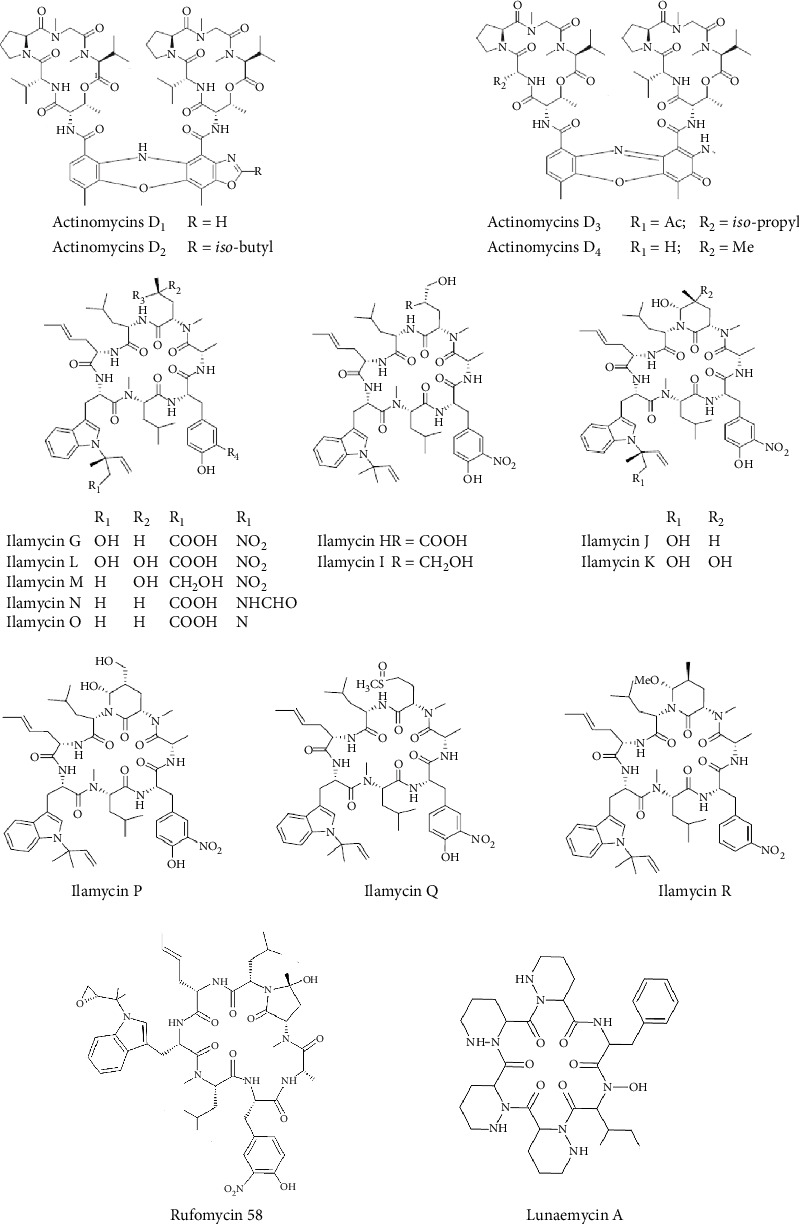
Chemical structures of cyclic peptides from *Streptomyces* with potent antibacterial activity against Gram-positive bacteria.

Over the past decade, several natural actinomycins have been described, notably those classified as D-type actinomycins. Among them, actinomycins D_1_–D_4_, derived from the fermentation broth of a strain of marine sponge-associated *Streptomyces* sp. LHW52447, demonstrated remarkable activity against Gram-positive bacteria (Figure 7). Specifically, actinomycins D_1_ and D_2_ exhibited potent antibacterial activity against MRSA (strain ATCC 33591), with MIC values ranging from 0.125 to 0.25 *μ*g/mL, compared to D_3_ and D_4_, which showed MIC values between 0.5 and 1.0 *μ*g/mL (Table 4) [70]. The enhanced activity of D_1_ and D_2_ suggests that the presence of an additional oxazole ring in the phenoxazinone chromophore markedly boosts anti-MRSA efficacy [70].

Additionally, ilamycins, also referred to as rufomycins, constitute a significant family of cycloheptapeptides for their antibacterial properties against Gram-positive bacteria (Figure 7). During antituberculosis drug discovery efforts, 12 new ilamycins were isolated from a large-scale (200 L) fermentation of the mutant strain *S. atratus* SCSIO ZH16 *ΔilaR*. These compounds demonstrated potent antitubercular activity against *Mycobacterium tuberculosis* H37Rv, with MIC values ranging from 0.001 to 1.058 *μ*g/mL (0.0096–10 *μ*M) (Table 4) [71]. SAR data point to two important features: cyclization at C-33 improves the antitubercular activity of ilamycins significantly, and the C-43 nitro group appears to play a key role in determining antitubercular activity [71].

Another potent anti-*M. tuberculosis* H37Rv rufomycin, rufomycin 58, was isolated from *S*. *atratus* strain MJM3502. It strongly inhibited this bacterium, with an MIC value of just 0.0088 *μ*g/mL (0.0085 *μ*M) (Table 4) [72]. It was concluded that the new in-chain cyclic 2-pyrrolidinone, in rufomycin 58, was linked to its potent anti-*M. tuberculosis* activity, comparable to or greater than that of the 2-piperidinone structures [72].

Lunaemycin A, a cyclic hexapeptide, was successfully isolated from the cave moonmilk-derived *S. lunaelactis* MM109T (Figure 7). It demonstrated efficacy in inhibiting a panel of bacteria, including *B*. *subtilis* ATCC 6633, *Streptococcus pyogenes* ATCC 12344, *S*. *aureus* (ATCC 25923 and ATCC 43300), *Staphylococcus epidermidis* SI-1266, *Staphylococcus haemolyticus* SI-6/2011, *Staphylococcus warneri* SI-5/2011, *E*. *faecalis* (ATCC 29212 and SI-759), and *E*. *faecium* SI-1831, with MICs of 0.12 and 0.25 *μ*g/mL (Table 4) [73].

##### 3.5.2.2. Lipopeptides

**Figure 8:**
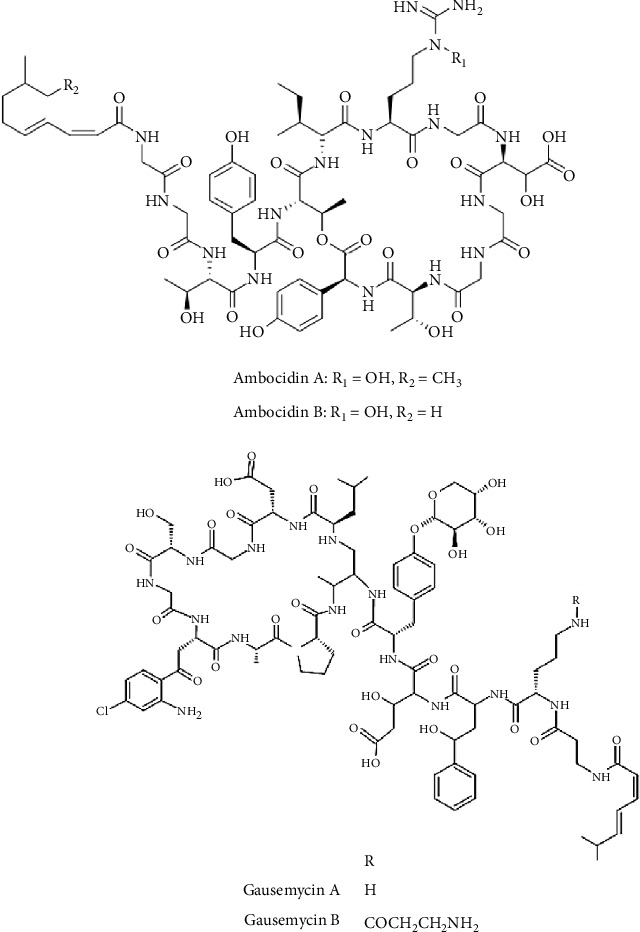
Chemical structures of lipopeptides from *Streptomyces* with potent antibacterial activity against Gram-positive bacteria.

Among the 15 identified lipopeptides, ambocidins A and B have emerged as the most potent, exhibiting significant inhibitory activity against Gram-positive bacteria (Figure 8 and Table 5). These compounds, identified as calcium-dependent lipodepsipeptides, were induced and isolated by cloning a silent BGC (the *amb* cluster) from *S. ambofaciens* ATCC 2387 and integrating it into the chromosome of *S. avermitilis*. Ambocidins A and B strongly inhibited *B. subtilis* E168 with an MIC value below 0.031 *μ*g/mL. Against other Gram-positive bacteria (*S*. *aureus* and *E. faecium*), ambocidin A remains the most active, with MIC values ranging from 0.25 to only 1 *μ*g/mL [88]. SAR observations reveal that both the fatty acid chain length and hydroxylation of Arg^7^ have a significant effect on activity, with ambocidin A emerging as the most effective congener [88].

Gausemycins A and B, two additional potent lipopeptides, were purified from *Streptomyces* sp. INA-Ac-5812 (Figure 8). These lipoglycopeptides exhibited strong antistaphylococcal activity against *S*. *aureus* ATCC 29213, *S*. *aureus* ATCC 33592 (MRSA), and *S*. *epidermidis* ATCC 14990, with MICs ranging from 0.25 to 1 *μ*g/mL and remained moderately to weakly active against other Gram-positive bacteria (Table 5) [89].

##### 3.5.2.3. RiPPs: Lassopeptides and Thiopeptides

Over the past decade, five RiPPs inhibiting exclusively Gram-positive bacteria have been identified, among which globimycin and sviceucin stand out as the most potent, demonstrating remarkable inhibitory activity (Table 6).

**Figure 9:**
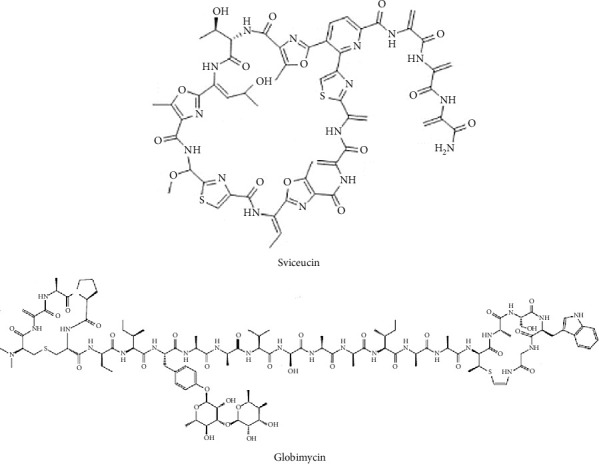
Chemical structures of RiPPs from *Streptomyces* with potent antibacterial activity against Gram-positive bacteria.

Globimycin, a thiopeptide, was discovered through genome mining from the extract of *S. globisporus* subsp. *globisporus* (Figure 9). This peptide exhibits strong antibacterial activity against *B. subtilis*, *S. aureus*, and *M. luteus*, with MIC values ranging from 0.25 to 1 *μ*g/mL (Table 6) [96].

Sviceucin, a lasso peptide, was isolated from the culture broth of *S. sviceus* (Figure 9). It selectively targets Gram-positive bacteria, including *Bacillus megaterium*, *Lactobacillus bulgaricus* 340, *S. aureus* subsp. *aureus* ATCC 6538, and *L. sakei* subsp. *sakei* DSM 20017, with MIC values ranging from 1.3 to 2.6 *μ*g/mL (1.25–2.5 *μ*M). Additionally, sviceucin was shown to inhibit *fsr* quorum sensing in *E. faecalis* (Table 6) [97].

#### 3.5.3. Terpenoids

**Figure 10:**
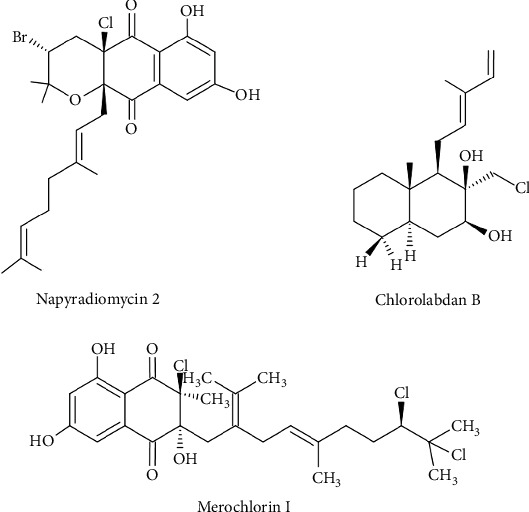
Chemical structures of terpenoids from *Streptomyces* with potent antibacterial activity against Gram-positive bacteria.

As shown in Table 7, 23 terpenoids, displaying antibacterial activity against Gram-positive bacteria, from *Streptomyces* have been isolated over the past decade. These compounds, although generally less effective than previously discovered compounds, include notable exceptions with significant antibacterial activity. Among them, napyradiomycin 2, belonging to the meroterpenoid class, stands out (Figure 10). Isolated from the culture broth of a marine-derived actinomycete (*Streptomyces* sp. SCSIO 10428), this compound exhibited significant antibacterial activity against *S. aureus* ATCC 29213, *B. subtilis* SCSIO BS01, and *B. thuringiensis* SCSIO BT01, with MIC values of 0.5, 1, and 1 *μ*g/mL, respectively (Table 7) [101]. Belonging to the same class of meroterpenoids, the newly identified merochlorin I exhibits potent antibacterial activity (Figure 10). It was purified, along with three other merochlorins, from the liquid culture of *Streptomyces* sp. CNH-189, a strain isolated from a marine sediment. Merochlorin I demonstrated significant antibacterial effect against *B. subtilis* KCTC 1021, *K. rhizophila* KCTC 1915, and *S. aureus* KCTC 1927, with MIC values of 1, 2, and 2 *μ*g/mL, respectively (Table 7) [102]. It is important to point out that the presence of a polar moiety at the isoprene chain (C-19) abolishes the antibacterial properties of merochlorins, which was shown with the hydroxy group in merochlorin H and the amine group in merochlorin J [102].

Additionally, chemical investigation of the ethyl acetate extract from the marine-derived *S. griseorubens* led to the discovery of five new labdane-type diterpenoids. Among them, chlorolabdan B exhibited significant activity, with MIC values ranging from 4 to 8 *μ*g/mL against *B. subtilis* KCTC 102, *M. luteus* KCTC 1915, and *S. aureus* KCTC 1927 (Table 7 and Figure 10) [103].

#### 3.5.4. Polyketides–Terpenoids

**Figure 11:**
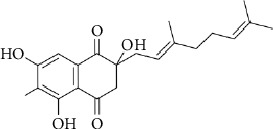
Chemical structure of flaviogeranin D.

Naphthoquinone-based meroterpenoids are a class of hybrid natural products derived from polyketide and terpenoid biosynthetic pathways. Since 2013, only five compounds have been isolated with exclusive activity against Gram-positive bacteria (Table 8). Among them, flaviogeranin D demonstrated significant antibacterial activity, with MIC values of 5.2 *μ*g/mL against *Mycobacterium smegmatis* MC^2^ 255 and 9.2 *μ*g/mL against *S*. *aureus* ATCC 43300 (Table 8 and Figure 11) [107]. Unlike its analogues (flaviogeranin B1 and flaviogeranin B) bearing an amine at C-8, the absence of this group in flaviogeranin D correlates with increased activity [107].

#### 3.5.5. Alkaloids

**Figure 12:**
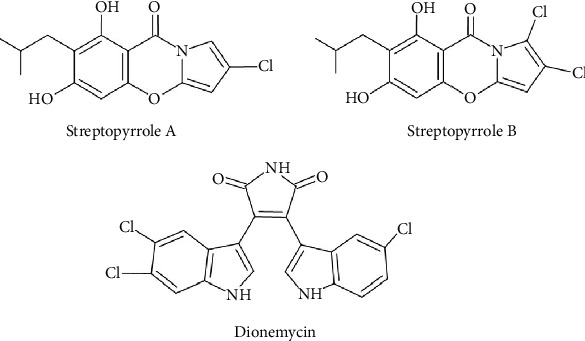
Chemical structures of alkaloids from *Streptomyces* with potent antibacterial activity against Gram-positive bacteria.

Among six alkaloids described, three of them stand out as the most potent, demonstrating remarkable inhibitory activity (Table 9). The first ones are streptopyrroles B and C, which were isolated from the marine-derived actinomycete, strain *S. zhaozhouensis* 208DD-064 (Figure 12). These pyrrole-containing alkaloids showed significant activity against *B*. *subtilis* KCTC 1021, *M*. *luteus* KCTC 1915, and *S*. *aureus* KCTC 1927 with MIC values ranging from 0.23 to 0.98 *μ*g/mL (0.7–2.9 *μ*M) (Table 9) [109]. Against *B*. *subtilis* KCTC 1021, streptopyrrole B is more potent (MIC: 0.26 *μ*g/mL [0.8 *μ*M]) than streptopyrrole C (MIC: 0.98 *μ*g/mL [2.9 *μ*M]). SAR suggests that monochloride substitution (streptopyrrole B) enhances potency compared to dichloride substitution (streptopyrrole C) [109]. The second one is dionemycin, a chlorinated *bis*-indole alkaloid obtained from deep-sea derived *Streptomyces* sp. SCSIO 11791 (Figure 12). It shows antistaphylococcal activity with an MIC range of 1–2 *μ*g/mL against clinic strains of MRSA and an MIC of 0.5 *μ*g/mL against *M*. *luteus* ML01 Ju1 (Table 9) [110]. The chlorine at C-6⁣^″^, in dionemycin, appears essential for increased antibacterial activity in contrast to similar analogues lacking this substitution [110].

#### 3.5.6. Phenazines

We present below four new phenazine metabolites discovered in the last decade exhibiting exclusive activity against Gram-positive bacteria (Table 10).

**Figure 13:**
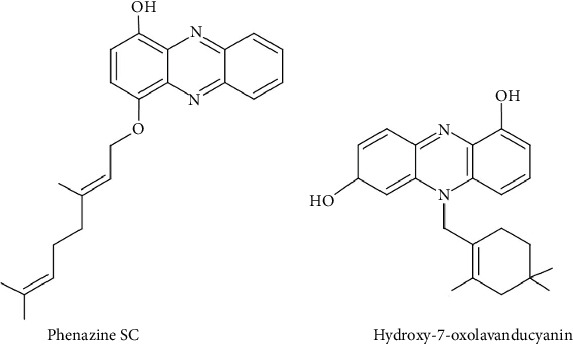
Chemical structures of phenazines from *Streptomyces* with potent antibacterial activity against Gram-positive bacteria.

The compounds phenazine SC and hydroxy-7-oxolavanducyanin have emerged as highly potent phenazines with remarkable inhibitory activity (Figure 13). Phenazine SC, derived from *Streptomyces* sp. (strain NA04227) associated with an earwig, demonstrated strong activity against *M. luteus*, with an MIC of 1.48 *μ*g/mL (Table 10) [112]. Its antibacterial strength is attributed to the presence of a geranyl group, which is not found in other related phenazine derivatives (SA and SB) [112].

Hydroxy-7-oxolavanducyanin, an analogue of lavanducyanin, was isolated from a soil-derived actinomycete (*Streptomyces* sp. CPCC 203577) (Figure 13). It exhibited significant activity against *S. epidermidis* ATCC 12228 (MSSE), *S. aureus* ATCC 29213 (MSSA), and *S. aureus* ATCC 33591, with MIC values of 0.06, 8, and 8 *μ*g/mL, respectively (Table 10) [113].

#### 3.5.7. Nucleoside Antibiotics

**Figure 14:**
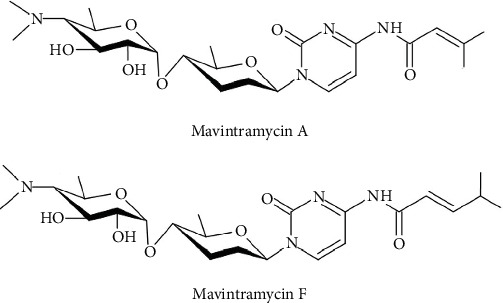
Chemical structures of nucleoside antibiotics from *Streptomyces* with potent antibacterial activity against Gram-positive bacteria.

Among 11 nucleoside antibiotics described, mavintramycins A and F demonstrate remarkable inhibitory activity (Table 11 and Figure 14). Mavintramycins A and F, isolated from the culture broth of *Streptomyces* sp. OPMA40551, were identified as active compounds against the *Mycobacterium avium* complex, specifically targeting both *M. avium* and *Mycobacterium intracellulare* (Figure 14). These compounds exhibited the most potent and selective inhibitory activity among the tested compounds, with MIC values ranging from 0.39 to 3.12 *μ*g/mL for mavintramycin A and 0.78–3.12 *μ*g/mL for mavintramycin F (Table 11) [116].

#### 3.5.8. Other Compounds

Over the past decade, 19 other novel compounds produced by *Streptomyces* species, specifically exhibiting activity against Gram-positive bacteria, have been identified (Table 12). These compounds belong to distinct chemical classes, with approximately 30% demonstrating moderate to weak activity depending on the target strain.

## 4. Discussion

In the years between 1940 and 1960, a period known as the golden age of antibiotic discovery, 20 new classes of antibiotics have been developed. However, with the beginning of the 21st century, only two new classes were marketed: oxazolidin-2-ones and daptomycin [129, 130]. Most of the antibiotics discovered during the antibiotic golden age are still used clinically, but their efficacy has been reduced by the emergence of multidrug-resistant bacteria [129, 131]. Among the problematic bacterial strains are Gram-positive bacteria such as *S*. *aureus*, *E. faecium*, *Streptococcus pneumoniae*, and *M*. *tuberculosis* [132]. The increasing reports of these strains resistant to first-line drugs [133] show that it is essential to develop new antibacterial compounds with core structures significantly different from those of the previous generation of antibiotics.

Over the past decade, *Streptomyces*-derived compounds have attracted increasing interest due to their notable bioactivity against Gram-positive bacteria, including antibiotic-resistant strains such as MRSA and VRE. This review highlighted 248 newly identified molecules, isolated from *Streptomyces* species or strains, belonging to different chemical classes. The polyketides and peptides are emerging as the most productive in terms of quantity and activity. This can be explained by the fact that between half and three-quarters of the BGCs in the *Streptomyces* genus encode enzyme complexes, such as NRPSs and PKSs, and their hybrid compounds [9].

Building on this molecular diversity, it is crucial to explore in more detail the biosynthetic mechanisms that allow *Streptomyces* to produce such structurally and functionally diverse substances. Among the compounds exhibiting considerable bioactivity, recent studies have elucidated or predicted biosynthetic pathways that highlight the metabolic flexibility of *Streptomyces*. Formicamycins, which are strong antibacterial aromatic polyketides derived from *S. formicae*, are produced through a type II PKS system. This pathway involves ketosynthases and cyclases that mediate the formation of aromatic polyketide scaffolds. Importantly, several enzymes that modify the structures post-PKS, such as halogenases and oxygenases, contribute to the structural diversity observed in formicamycins and fasamycins. The regulation of this BGC is carefully controlled by various transcription factors, making it a model for exploring the regulation of antibiotic production [32].

Ilamycins, also known as rufomycins, are complex cyclic peptides produced by bacteria using a specific biosynthetic pathway. A prenyltransferase enzyme modifies tryptophan by adding a prenyl group, resulting in the prenylated tryptophan that is part of the final molecule. Another key part of the pathway is a nitric oxide synthase (NOS) and a cytochrome P450 enzyme known as RufO. The NOS produces nitric oxide, and RufO uses this to nitrate tyrosine, forming 3-nitrotyrosine. This modified tyrosine is subsequently incorporated into the peptide by the NRPS. Once the ring is assembled, additional modifying enzymes, including other P450s, make further chemical transformations to complete the biosynthesis of ilamycins. This pathway enables bacteria, such as *Streptomyces*, to produce a range of biologically active compounds with strong antituberculosis properties [134]. These comprehensive biosynthetic insights not only highlight the enzymatic capacity of *Streptomyces* but also emphasize the link between metabolic pathways and their end-products.

Furthermore, SAR studies revealed critical insights about the structural features that affect the antibacterial effectiveness of newly identified *Streptomyces*-derived compounds. For instance, the patterns of halogenation, including the presence and type of halogen atoms (e.g., chlorine compared to bromine) in formicamycins and streptopyrroles, significantly influenced their antibacterial activity. Similarly, functional groups such as the 5-*O*-methyl in streptovertimycin G, or the oxazole ring in actinomycins D_1_ and D_2_, were found to boost activity when compared to their analogues (actinomycins D_3_ and D_4_). The importance of particular groups, such as the C-43 nitro group in ilamycins, the methyl group at C-2 in anthracimycin, and sugar substitutions at C-9 in lobophorins, were all identified as key factors for bioactivity. These insights into the SAR of *Streptomyces*-derived metabolites pave the way for a more detailed investigation of certain compounds, which not only dominate in their varied structures but also show remarkable antibacterial potency.

Focusing more specifically on polyketides, the aromatic subclass stands out not only as the most dominant but also as one of the most potent. Compounds such as cervinomycins and kebanmycins have demonstrated interestingly low MIC values (0.008 *μ*g/mL for cervinomycins B_4_ against *S. aureus* 16-30 [MRSA]) (Table 1). Cervinomycins B_1_–B_4_ are more efficient than cervinomycins A_1_ and A_2_, which were first isolated by *S. cervinus* sp. nov. [135] underlining their future therapeutic potential. In addition to these compounds, anthracimycin stands out as another powerful polyketide belonging to the macrolide subclass with a low minimum MIC value of 0.031 *μ*g/mL against *B. anthracis* (strain UM23C1-1) and other Gram-positive bacteria including *S. aureus* ATCC 13709 and *E. faecalis* ATCC 29212 (MICs: 0.0625–0.125 *μ*g/mL) (Table 2) [51]. While the majority of macrolide antibiotics act by targeting the 50S ribosomal subunit to block protein synthesis [136], it has been suggested that anthracimycin exhibits its bactericidal activity by inhibiting DNA and/or RNA synthesis in the absence of DNA intercalation [137]. Anthracimycin's high antibacterial potency, novel molecular structure, and new mechanism of action make it a target of choice for the development of new antibiotics [138].

Peptide-based antibiotics, notably actinomycins D_1_–D_4_ and ilamycins, possess strong anti-Gram-positive bacteria activity (Table 4). Actinomycins D_1_–D_4_ are structurally similar to actinomycin D, also called dactinomycin, which was the first antibiotic compound shown to have anticancer activity [87]. There are generally some structural differences between actinomycin compounds, which contribute to improving their antimicrobial profiles, confirming that actinomycins D_1_–D_4_ would probably be promising candidates for drug development, although further information on toxicity is required. Ilamycins also showed especial antituberculosis activity, such as rufomycin 58 with an MIC of just 0.0088 *μ*g/mL (Table 4). This suggests that certain peptide antibiotics may have dual activity against both common and mycobacterial pathogens. Previous studies have shown that ilamycins exert their antimycobacterial activity by inhibiting the proteolytic activity of the ClpC1/P1/P2 complex, a critical component involved in regulated protein degradation in *M. tuberculosis* [139].

In addition to these compounds, the recently discovered lipodepsipeptides ambocidins A and B have shown to be particularly effective as peptide-based antibiotics. These calcium-dependent cyclic lipopeptides demonstrated strong antibacterial activity against Gram-positive bacteria, with MIC values below 0.031 *μ*g/mL against *B. subtilis* E168 and MICs ranging from 0.25 to 1 *μ*g/mL against *S. aureus* and *E. faecium* (Table 5) [88]. In terms of their mode of action, ambocidins act by inhibiting bacterial cell wall biosynthesis through specific targeting of lipid II, a key precursor in the peptidoglycan synthesis pathway. Importantly, unlike vancomycin, ambocidins bind to a different site on lipid II, rather than relying on the D-Ala-D-Ala motif. This unique interaction was evidenced by their retained activity against VREfm and the failure of a D-Ala-D-Ala mimic to inhibit their activity [88]. These findings position ambocidins as promising candidates for the treatment of drug-resistant Gram-positive bacteria due to their strong activity and novel mechanism of action.

Despite terpenoids being less numerous, certain compounds such as napyradiomycin 2 and merochlorin I have demonstrated important antibacterial activity (MICs: 0.5–2 *μ*g/mL) (Table 7) [101, 102]. Historically, *Streptomyces* has been viewed as a limited source of terpenoids (with the exception of geosmin) [26]. However, recent findings suggest an untouched biosynthetic potential that could be revealed by genome exploration and the activation of silent gene clusters.

Although other classes such as alkaloids, phenazines, and nucleoside antibiotics may be numerically underrepresented, they contain compounds of significant potency. For example, streptopyrroles (A and B) and dionemycin, both alkaloids, displayed MICs below 2 *μ*g/mL (Table 9), highlighting the valuable bioactivity contributed by these less abundant chemical classes. Similarly, nucleoside antibiotics show significant antimicrobial potential, as highlighted by mavintramycin A (Table 11). This compound was discovered to be effective against the *M. avium* complex, specifically targeting both *M. avium* and *M. intracellulare.* When examining its main mode of action, mavintramycin A completely inhibited protein synthesis and strongly inhibited DNA synthesis in *M. smegmatis*, while it had very weak effects on RNA and peptidoglycan syntheses. Mavintramycin A was considered to bind to a distinct site on the ribosome compared to the commonly used clarithromycin and amikacin, corroborating the finding that no cross-resistant *M. avium* strains with this compound were identified [116].

In this systematic review, the majority of isolated compounds were mainly derived from marine environments (e.g., deep-sea sediments) and soil. This result is in line with the studies by Lacey and Rutledge [16] and Donald et al. [140]. This reinforces the idea that extreme ecosystems, like marine environments, are promising reservoirs of novel bioactive metabolites, possibly due to evolutionary pressure for chemical defense. Additionally, approximately 23% of these active compounds exhibited a range of additional bioactivities, including cytotoxic, antiviral, and neuroactive effects. This multifunctionality implies the presence of structural scaffolds with pharmacological versatility, potentially amenable to optimization for dual therapeutic uses.

Despite the promising, *in vitro*, antibacterial activity of the 248 reported compounds, only a small few (*n* = 5) have progressed to *in vivo* experiments, such as mavintramycin A, anthracimycin, and three liposidomycin congeners. These compounds have proven to be effective in animal models, particularly against infections caused by *M. avium* complex and *B. anthracis*.

In conclusion, this analysis underscores the significant role of *Streptomyces* as a rich source of structurally diverse and biologically potent compounds effective against Gram-positive bacteria. These compounds hold the potential to be developed into effective drugs to combat antibacterial resistance. The study also emphasizes the necessity of further exploring underrepresented environments and leveraging modern genetic tools to uncover hidden biosynthetic pathways.

The present systematic review has certain limitations. Firstly, out of the total selected articles, 20 could not be analyzed due to restricted access. In addition, several publications were excluded because they reported antibacterial activity using noncomparable metrics, such as DIZ, IC_50_, MIC_50_, MIC_90_, or MIC_99_, instead of standardized MIC values. This made it more difficult to select and present the most potent compounds. Despite these limitations, the defined inclusion and exclusion criteria, along with the consistent application of MIC-based potency assessments, contributed to a representative synthesis of the most promising compounds derived from the *Streptomyces* genus.

## 5. Conclusion

This systematic review summarizes *Streptomyces* anti-Gram-positive bacteria compounds reported in the last decade. The *Streptomyces* species/strains investigated originate from a wide range of biotopes, with marine sources being the most explored, followed by terrestrial soil. The recent investigations and efforts have led to the discovery of a total of 248 secondary metabolites representing diverse chemical classes, including polyketides, peptides, terpenoids, phenazines, and other natural products. The major ones are polyketides and peptides, representing 40.32% and 29.03% of the identified compounds. These discoveries show that *Streptomyces* remains an unexpectedly resource of structurally diverse natural products that can be developed into pharmaceutical drugs and, therefore, reduce multidrug resistance in Gram-positive bacteria, which poses a serious threat to human health.

## Figures and Tables

**Table 1:** Aromatic polyketide products with anti-Gram-positive bacteria activity.

**Compound**	***Streptomyces* species/strain**	**Source**	**Tested Gram-positive bacteria**	**MIC (*μ*g/mL)**	**Reference**
Cervinomycin B_1_	*Streptomyces* CPCC 204980	Soil	*S. aureus* ATCC 33591 (MRSA)	0.06	[30]
			*S. aureus* 16-30 (MRSA)	0.12	
			*Enterococcus faecalis* ATCC 51299 vancomycin resistant (VRE)	0.12	
			*E. faecalis* ATCC 51575 (VRE)	0.12	
			*Enterococcus faecium* ATCC 700221 (VRE)	0.06	
			*E. faecium* 12-1 (VRE)	0.12	
Cervinomycin B_2_			*S. aureus* ATCC 33591 (MRSA)	0.25	
			*S. aureus* 16-30 (MRSA)	0.12	
			*E. faecalis* ATCC 51299 (VRE)	0.25	
			*E. faecalis* ATCC 51575 (VRE)	0.5	
			*E. faecium* ATCC 700221 (VRE)	0.5	
			*E. faecium* 12-1 (VRE)	0.25	
Cervinomycin B_3_			*S. aureus* ATCC 33591 (MRSA)	0.06	
			*S. aureus* 16-30 (MRSA)	0.06	
			*E. faecalis* ATCC 51299 (VRE)	0.06	
			*E. faecalis* ATCC 51575 (VRE)	0.12	
			*E. faecium* ATCC 700221 (VRE)	0.12	
			*E. faecium* 12-1 (VRE)	0.03	
Cervinomycin B_4_			*S. aureus* ATCC 33591 (MRSA)	0.016	
			*S. aureus* 16-30 (MRSA)	0.008	
			*E. faecalis* ATCC 51299 (VRE)	0.016	
			*E. faecalis* ATCC 51575 (VRE)	0.016	
			*E. faecium* ATCC 700221 (VRE)	0.03	
			*E. faecium* 12-1 (VRE)	0.03	

Kebanmycin A	*Streptomyces* sp. SCSIO 40068	Mangrove rhizosphere	*S. aureus* ATCC 29213	0.125	[31]
			MRSA shhs-A1	0.125	
			MRSA 1862	0.125	
			MRSA 669	0.5	
			MRSA 991	0.5	
			*B. subtilis* 1064	> 64	
Kebanmycin B			*S. aureus* ATCC 29213	2	
			MRSA shhs-A1	2	
			MRSA 1862	1	
			MRSA 669	2	
			MRSA 991	2	
			*B. subtilis* 1064	1	
Kebanmycin C			*S. aureus* ATCC 29213	0.5	
			MRSA shhs-A1	0.5	
			MRSA 1862	1	
			MRSA 669	2	
			MRSA 991	2	
			*B. subtilis* 1064	4	
Kebanmycin D			*S. aureus* ATCC 29213	32	
			MRSA shhs-A1	16	
			MRSA 1862	16	
			MRSA 669	16	
			MRSA 991	16	
			*B. subtilis* 1064	> 64	

Fasamycin C	*S. formicae*	Plant endophyte	*B. subtilis*	< 10 (< 20 *μ*M)	[32]
			*S. aureus* (MRSA)	20 (40 *μ*M)	
			*E. faecium* (VRE)	20 (40 *μ*M)	
Fasamycin D			*B. subtilis*	5.52 (10 *μ*M)	
			*S. aureus* (MRSA)	5.52 (10 *μ*M)	
			*E. faecium* (VRE)	5.52 (10 *μ*M)	
Fasamycin E			*B. subtilis*	2.94 (5 *μ*M)	
			*S. aureus* (MRSA)	47.04 (80 *μ*M)	
			*E. faecium* (VRE)	47.04 (80 *μ*M)	
Formicamycin A			*B. subtilis*	2.75 (5 *μ*M)	
			*S. aureus* (MRSA)	> 44.12 (> 80 *μ*M)	
			*E. faecium* (VRE)	> 44.12 (> 80 *μ*M)	
Formicamycin B			*B. subtilis*	5.73 (10 *μ*M)	
			*S. aureus* (MRSA)	5.73 (10 *μ*M)	
			*E. faecium* (VRE)	5.73 (10 *μ*M)	
Formicamycin C			*B. subtilis*	2.93 (5 *μ*M)	
			*S. aureus* (MRSA)	0.73 (1.25 *μ*M)	
			*E. faecium* (VRE)	46.88 (80 *μ*M)	
Formicamycin D			*B. subtilis*	6.07 (10 *μ*M)	
			*S. aureus* (MRSA)	12.15 (20 *μ*M)	
			*E. faecium* (VRE)	6.07 (10 *μ*M)	
Formicamycin E			*B. subtilis*	6.21 (10 *μ*M)	
			*S. aureus* (MRSA)	12.43 (20 *μ*M)	
			*E. faecium* (VRE)	6.21 (10 *μ*M)	
Formicamycin F			*B. subtilis*	3.10 (5 *μ*M)	
			*S. aureus* (MRSA)	12.43 (20 *μ*M)	
			*E. faecium* (VRE)	1.55 (2.5 *μ*M)	
Formicamycin G			*B. subtilis*	3.10 (5 *μ*M)	
			*S. aureus* (MRSA)	—	
			*E. faecium* (VRE)	—	
Formicamycin H			*B. subtilis*	6.21 (10 *μ*M)	
			*S. aureus* (MRSA)	—	
			*E. faecium* (VRE)	—	
Formicamycin I			*B. subtilis*	< 1.6 (< 2.5 *μ*M)	
			*S. aureus* (MRSA)	< 1.6 (< 2.5 *μ*M)	
			*E. faecium* (VRE)	0.80 (1.25 *μ*M)	
Formicamycin J			*B. subtilis*	< 13.12 (< 20 *μ*M)	
			*S. aureus* (MRSA)	0.41 (0.625 *μ*M)	
			*E. faecium* (VRE)	0.82 (1.25 *μ*M)	
Formicamycin K			*B. subtilis*	< 1.63 (< 2.5 *μ*M)	
			*S. aureus* (MRSA)	1.63 (2.5 *μ*M)	
			*E. faecium* (VRE)	3.26 (5 *μ*M)	
Formicamycin L			*B. subtilis*	< 1.75 (< 2.5 *μ*M)	
			*S. aureus* (MRSA)	0.87 (1.25 *μ*M)	
			*E. faecium* (VRE)	1.75 (2.5 *μ*M)	

Streptovertimycins A–C, E, and F	*S. morookaense*	Soil	*S. aureus* 11646 (MRSA)	2.5	[33]
			*S. aureus* ATCC 6548 methicillin-sensitive (MSSA)	2.5	
			*E. faecium* (VRE)	2.5	
			*E. faecium* vancomycin-sensitive (VSE)	2.5	
Streptovertimycin D			*S. aureus* 11646 (MRSA)	1.25	
			*S. aureus* ATCC 6548 (MSSA)	1.25	
			*E. faecium* (VRE)	1.25	
			*E. faecium* (VSE)	1.25	
Streptovertimycin G			*S. aureus* 11646 (MRSA)	0.63	[33]
			*S. aureus* ATCC 6548 (MSSA)	0.63	
			*E. faecium* (VRE)	1.25	
			*E. faecium* (VSE)	1.25	
Streptovertimycin H			*S. aureus* 11646 (MRSA)	5	
			*S. aureus* ATCC 6548 (MSSA)	5	
			*E. faecium* (VRE)	5	
			*E. faecium* (VSE)	5	

Mzabimycin A	*Streptomyces* sp. PAL114	Soil	*Bacillus subtilis* ATCC 6633	50	[34]
			*Micrococcus luteus* ATCC 9314	15	
			*S. aureus* MRSA 639c	60	
			*S. aureus* S1	80	
			*Listeria monocytogenes* ATCC 13932	40	
Mzabimycin B			*B. subtilis* ATCC 6633	50	
			*M. luteus* ATCC 9314	15	
			*S. aureus* MRSA 639c	60	
			*S. aureus* S1	80	
			*L. monocytogenes* ATCC 13932	20	

Grincamycin L	*S. lusitanus* OUCT16-27	Marine sediment	*E. faecalis* CCARM 5172	6.25	[35]
			*E. faecium* CCARM 5203	3.12	
			*S. aureus* CCARM 3090	6.25	

Marangucyclines A and B	*Streptomyces* sp. SCSIO 11594	Marine sediment	*E. faecalis* ATCC29212	64	[36]

Angumycinone A	*Streptomyces* sp. KMC004	Acidic mine drainage	*M. luteus* KCCM1548	6.25	[37]
			*E. hirae* KCCM11768	12.5	
			*S. aureus* CCARM3089 MRSA	—	
Angumycinone B			*M. luteus* KCCM1548	0.78	
			*Enterococcus hirae* KCCM11768	1.56	
			*S. aureus* CCARM3089 MRSA	12.5	

Pratensilin D	*Streptomyces* sp. KCB-132	Marine sediment	*Bacillus cereus* CMCC 32210	4	[38]

Angucycline 15	*Streptomyces* sp. CB01913	Soil	*S*. *aureus* ATCC 25923	11	[39]
			*B*. *subtilis* ATCC 23857	8.1	
			*Mycobacterium smegmatis* ATCC 607	9.7	
Angucycline 16			*S*. *aureus* ATCC 25923	> 100	
			*B*. *subtilis* ATCC 23857	25	
			*M. smegmatis* ATCC 607	> 120	
Angucyclinone 5			*S*. *aureus* ATCC 25923,	> 100	
			*B*. *subtilis* ATCC 23857	85	
			*M*. *smegmatis* ATCC 607	93	

Urdamycin W	*S. ardesiacus* 156VN-095	Sponge	*B*. *subtilis* KCTC 1021	8	[40]
			*M. luteus* KCTC 1915	64	
			S. *aureus* KCTC 1927	32	
Urdamycin X			*B*. *subtilis* KCTC 1021	> 128	
			*M. luteus* KCTC 1915	64	
			S. *aureus* KCTC 1927	64	
Grincamycin U			*B*. *subtilis* KCTC 1021	32	
			*M. luteus* KCTC 1915	> 128	
			S. *aureus* KCTC 1927	> 128	

Steffimycin E	*Streptomyces* sp. OPMA02852	Marine	*Mycobacterium intracellulare* JCM6384	6.25	[41]
			*M. smegmatis* M341	12.5	
			*Mycobacterium bovis* BCG Pasteur	25	
			*Mycobacterium avium* JCM15430	> 50	

Isotirandamycin B	*Streptomyces* sp. SCSIO 41399	Coral	*Streptococcus agalactiae*	5	[42]
			*S. aureus*	> 50	

Misamycin	*Streptomyces* sp. YIM66403	Plant endophyte	*S. aureus*	64	[43]

N-acetyl-N demethylmayamycin	*Streptomyces* sp. 182SMLY	Marine sediment	*S. aureus* ATCC 43300	10.18 (20 *μ*M)	[44]

Accramycin A	*Streptomyces* sp. MA37	Plant rhizosphere	*Streptococcus* B. ATCC 12386	27	[45]

Diepoxyactinorhodin	*Streptomyces* sp. strain MC11141	Soil	*S. aureus* (MRSA)	32	[46]
			*S. aureus* (MSSA)	16	
			*B. subtilis*	8	

Zunyimycin B	*Streptomyces* sp. FJS31-2	Soil	*S. aureus* ATCC 29213	3.94	[47]
			*S. aureus* MRSA 08301	7.88	
			*S. aureus* MRSA 161222330	25.62	
			*S. aureus* MRSA 161231380	12.81	
			*S. aureus* MRSA 170108317	25.62	
			*S. aureus* MRSA 161231350	25.62	
			*E. faecalis* ATCC 29212	15.75	
			*E. faecalis* 160803348	12.81	
			*E. faecalis* 160804314	12.81	
			*E. faecalis* 161222328	12.81	
			*E. faecalis* 170106034	25.62	
			*B. subtilis* CGMCC 1.2428	15.75	
Zunyimycin C			*S. aureus* ATCC 29213	0.94	
			*S. aureus* MRSA 08301	3.75	
			*S. aureus* MRSA 161222330	8.14	
			*S. aureus* MRSA 161231380	4.07	
			*S. aureus* MRSA 170108317	4.07	
			*S. aureus* MRSA 161231350	4.07	
			*E. faecalis* ATCC 29212	7.50	
			*E. faecalis* 160803348	4.07	
			*E. faecalis* 160804314	8.14	
			*E. faecalis* 161222328	4.07	
			*E. faecalis* 170106034	8.14	
			*B. subtilis* CGMCC 1.2428	3.75	

AN483	*Streptomyces* sp. AN100483	Soil	*S*. *aureus* RN4220	32	[48]
			*S*. *aureus* CCARM 3167		
			*S*. *aureus* CCARM 3506		
			*S*. *aureus* CCARM 3505		
			*S*. *aureus* CCARM 3519		

Isofuranonaphthoquinone A	*Streptomyces* sp. CB01883	Mutant	*S. aureus* ATCC 25923	86.7	[49]
			*B. subtilis* ATCC 23857	> 100	
			*M. smegmatis* ATCC 607	> 100	
Isofuranonaphthoquinones E and F			*S. aureus* ATCC 25923	58	
			*B. subtilis* ATCC 23857	58	
			*M. smegmatis* ATCC 607	72.5	
Isofuranonaphthoquinone G			*S. aureus* ATCC 25923	75.6	
			*B. subtilis* ATCC 23857	> 100	
			*M. smegmatis* ATCC 607	94.5	

**Table 2:** Macrolide polyketide products with anti-Gram-positive bacteria activity.

**Compound**	***Streptomyces* species/strain**	**Source**	**Tested Gram-positive bacteria**	**MIC (*μ*g/mL)**	**Reference**
Anthracimycin	*Streptomyces* sp. CNH365	Marine sediment	*Bacillus anthracis* UM23C1-1	0.03125	[51]
			*S. aureus* ATCC 13709	0.0625	
			*E. faecalis* ATCC 29212	0.125	

Anthracimycin B	*S. cyaneofuscatus* M-169	Coral	*S. aureus* MB5393 (MRSA)	0.125–0.25	[52]
			*S. aureus* ATCC 29213 (MSSA)	4–8	
			*E. faecium* CL144754 (VSE)	0.125–0.25	
			*E. faecalis* CL144492 (VSE)	0.25–0.5	

Lobophorin L	*Streptomyces* sp. 4506	Marine	*M*. *luteus* ATCC 10240	8	[53]
			*Bacillus thuringiensis* BT01	4	
			*S*. *aureus* ATCC 29213	> 128	
			*S*. *aureus* ATCC 43300 (MRSA)	> 128	
Lobophorin M			*M*. *luteus* ATCC 10240	> 128	
			*B. thuringiensis* BT01	> 128	
			*S*. *aureus* ATCC 29213	> 128	
			*S*. *aureus* ATCC 43300 (MRSA)	> 128	

Auroramycin	*S. roseosporus* NRRL 15998	Unknown	*S. aureus* (MRSA) clinical N216	1–2	[54]
			*S. aureus* (VI-MRSA) clinical Z172	2–4	
			*E. faecalis* (VRE) ATCC 51299	1	

Borrelidins J and K	*Streptomyces* sp. NA06554	Plant endophyte	*M. luteus* CMCC(B) 28,001	> 126.25 (> 250 *μ*M)	[55]
			*S. aureus* CMCC(B) 26003	> 126.25 (> 250 *μ*M)	
			*B*. *subtilis* CICC 10283	> 126.25 (> 250 *μ*M)	
Borrelidin L			*M. luteus* CMCC(B) 28,001	20.2 (40 *μ*M)	
			*S. aureus* CMCC(B) 26003	> 126.25 (> 250 *μ*M)	
			*B*. *subtilis* CICC 10283	> 126.25 (> 250 *μ*M)	

Aldgamycins J and L	*Streptomyces* sp. HK-2006-1	Marine sediment	*S. aureus* 209P	500	[56]
Aldgamycin K				> 1000	
Aldgamycins M and N				32	
Aldgamycin O				16	

Lobophorin H	*Streptomyces* sp. 12A35	Marine sediment	*S. aureus* ATCC 29213	50	[57]
			*B. subtilis* CMCC 63501	1.57	
Lobophorin I			*S. aureus* ATCC 29213	> 100	
			*B. subtilis* CMCC 63501	50	

Lobophorin G	*Streptomyces* sp. MS100061	Marine sediment	*M. bovis* BCG	1.56	[58]
			*Mycobacterium tuberculosis* H37Rv	32	
			*B. subtilis* ATCC 6663	3.125	
			*S. aureus*	> 50	
			*S*. *aureus* MRSA	> 50	

13-Deoxytetrodecamycin	*Streptomyces* sp. WAC04657	Unknown	*B*. *subtilis* 168	2	[59]
			*M*. *luteus*	8	
			*S. aureus* ATCC 29213	8	
			*S*. *aureus* ATCC BAA-41	1	
			*S*. *aureus* ATCC BAA-44	8	
			*Staphylococcus saprophyticus* ATCC 15305	8	
			*Staphylococcus epidermidis* ATCC 12228	8	
			*E*. *faecalis* ATCC 29212	4	

Borrelidin M	*S. olivaceus* SCSIO LO13	Mollusk endophyte	*M*. *luteus*	16.8 (33 *μ*M)	[60]
			*B. subtilis*	66.69 (131 *μ*M)	
			*E. faecalis* ATCC 29212	> 101.18 (> 200 *μ*M)	
			*S*. *aureus* ATCC 29213	> 101.18 (> 200 *μ*M)	
Borrelidin N			*M*. *luteus*	> 107.06 (> 200 *μ*M)	
			*B. subtilis*	> 107.06 (> 200 *μ*M)	
			*E. faecalis* ATCC 29212	> 107.06 (> 200 *μ*M)	
			*S*. *aureus* ATCC 29213	> 107.06 (> 200 *μ*M)	
Borrelidin O			*M*. *luteus*	> 101 (> 200 *μ*M)	
			*B. subtilis*	> 101 (> 200 *μ*M)	
			*E. faecalis* ATCC 29212	> 101 (> 200 *μ*M)	
			*S*. *aureus* ATCC 29213	> 101 (> 200 *μ*M)	

Tylosin Analogue 1	*S. ansochromogenes* 7100 (*Δ*wblA) *S.*	Mutant	*Streptococcus pneumoniae*	7.06	[61]
			*Streptococcus pyogenes*	3.53	
			*S. epidermidis*	> 100	
			*S. aureus* CGMCC1.89	56.5	
			*B. subtilis* CGMCC1.1630	14.1	
			*B. cereus* CGMCC1.1626	28.2	
Tylosin Analogue 2			*S. pneumoniae*	7.31	
			*S. pyogenes*	3.65	
			*S. epidermidis*	> 100	
			*S. aureus* CGMCC1.89	58.5	
			*B. subtilis* CGMCC1.1630	14.6	
			*B. cereus* CGMCC1.1626	29.2	

Neoabyssomicins F and G	*S*. *koyangensis* SCSIO 5802	Marine sediment	*S*. *aureus* 29213	32	[62]
			*S*. *aureus* MRSA 669	16	
			*S*. *aureus* MRSA 1862	16	
			*S*. *aureus* MRSA shhs-A1	16	
			*E*. *faecalis* ATCC 29212	32	

Damavaricin H	*S*. *spectabilis* CCTCC M2017417	Mutant	MRSA ATCC 43300	4	[63]
			MRSA USA300 LAC	8	
			MRSA USA400 MW2	8	
Protostreptovaricin VI			MRSA ATCC 43300	8	
			MRSA USA300 LAC	16	
			MRSA USA400 MW2	16	

23-O-Butyrylbafilomycin D	*Streptomyces* sp. HZP-2216E	Algae endophyte	*S*. *aureus* (MRSA)	5.16 (7.4 *μ*M)	[64]

21,22-En-bafilomycin D	*Streptomyces* sp. HZP-2216E	Algae endophyte	*S*. *aureus* (MRSA)	12.5	[65]
21,22-En-9-hydroxybafilomycin D				12.5	

**Table 3:** Other polyketide products with anti-Gram-positive bacteria activity.

**Compound**	***Streptomyces* species/strain**	**Source**	**Tested Gram-positive bacteria**	**MIC (*μ*g/mL)**	**Reference**
Violapyrone A	*S. violascens*	Hylobates hoolock	*B. subtilis* ATCC 6633	32	[67]
			*S. aureus* ATCC 25923	8	
Violapyrone B			*B. subtilis* ATCC 6633	4	
			*S. aureus* ATCC 25923	4	
Violapyrone C			*B. subtilis* ATCC 6633	16	
			*S. aureus* ATCC 25923	16	
Violapyrones D and G			*B. subtilis* ATCC 6633	—	
			*S. aureus* ATCC 25923	128	
Violapyrone E			*B. subtilis* ATCC 6633	128	
			*S. aureus* ATCC 25923	128	

Chresdihydrochalcone	*S. chrestomyceticus* BCC 24770	Soil	*S. aureus* ATCC 44300 (MRSA)	25	[68]
			*M. luteus* ATCC 10040	25	
			*S*. *aureus* ATCC 25923	18	
			*B. subtilis* JH642	10	

Chrysomycin F	*Streptomyces* sp. MS751	Marine sediment	*M*. *bovis* BCG	50	[69]
			*M*. *tuberculosis* H37Rv	> 100	
			*M*. *tuberculosis* (Hr1)	> 100	
			*M*. *tuberculosis* (Hr2)	> 100	
			*M*. *tuberculosis* (Hr3)	> 100	
			*M*. *tuberculosis* (Hr4)	> 100	
			*M*. *tuberculosis* (Hr5)	> 100	
			*M*. *smegmatis* mc2155	> 100	
			*S*. *aureus* (MRSA)	> 25	
			*S*. *aureus* ATCC 6538	> 25	
			*S*. *pneumoniae* ATCC 49619	> 100	
Chrysomycin G			*M*. *bovis* BCG	12.5	
			*M*. *tuberculosis* H37Rv	50	
			*M*. *tuberculosis* (Hr1)	> 100	
			*M*. *tuberculosis* (Hr2)	> 100	
			*M*. *tuberculosis* (Hr3)	> 100	
			*M*. *tuberculosis* (Hr4)	> 100	
			*M*. *tuberculosis* (Hr5)	> 100	
			*M*. *smegmatis* mc2155	> 100	
			*S*. *aureus* (MRSA)	> 100	
			*S*. *aureus* ATCC 6538	> 100	
			*S*. *pneumoniae* ATCC 49619	50	
Chrysomycins H–J			*M*. *bovis* BCG	> 100	
			*M*. *tuberculosis* H37Rv	> 100	
			*M*. *tuberculosis* (Hr1)	> 100	
			*M*. *tuberculosis* (Hr2)	> 100	
			*M*. *tuberculosis* (Hr3)	> 100	
			*M*. *tuberculosis* (Hr4)	> 100	
			*M*. *tuberculosis* (Hr5)	> 100	
			*M*. *smegmatis* mc2155	> 100	
			*S*. *aureus* (MRSA)	> 100	
			*S*. *aureus* ATCC 6538	> 100	
			*S*. *pneumoniae* ATCC 49619	> 100	

**Table 4:** Cyclic peptide products with anti-Gram-positive bacteria activity.

**Compound**	***Streptomyces* species/strain**	**Source**	**Tested Gram-positive bacteria**	**MIC (*μ*g/mL)**	**Reference**
Actinomycin D_1_	*Streptomyces* sp. LHW52447	Sponge	*S. aureus* P172 (MRSA)	0.125	[70]
			*S. aureus* P175 (MRSA)	0.25	
			*S. aureus* ATCC 33591 (MRSA)	0.125	
Actinomycin D_2_			*S. aureus* P172 (MRSA)	0.25	
			*S. aureus* P175 (MRSA)	0.25	
			*S. aureus* ATCC 33591 (MRSA)	0.25	
Actinomycin D_3_			*S. aureus* P172 (MRSA)	0.5	
			*S. aureus* P175 (MRSA)	1	
			*S. aureus* ATCC 33591 (MRSA)	0.5	
Actinomycin D_4_			*S. aureus* P172 (MRSA)	0.5	
			*S. aureus* P175 (MRSA)	0.25	
			*S. aureus* ATCC 33591 (MRSA)	0.25	

Ilamycins G and H	*S*. *atratus* SCSIO ZH16	Marine sediment	*M*. *tuberculosis* H37Rv	1.005 (9.5 *μ*m)	[71]
Ilamycins I and R				0.12 (1.2 *μ*M)	
Ilamycin J				0.001 (0.0096 *μ*M)	
Ilamycins K, P, and Q				0.148 (1.4 *μ*M)	
Ilamycin L				0.025 (0.24 *μ*M)	
Ilamycin M				0.24 (2.3 *μ*M)	
Ilamycin N				1.016 (9.6 *μ*M)	
Ilamycin O				1.058 (10 *μ*M)	

Rufomycin 58	*S*. *atratus* MJM3502	Unknown	*M*. *tuberculosis* H37Rv	0.0088 (0.0085 *μ*M)	[72]
			*Mycobacterium abscessus*	1.24 (1.2 *μ*M)	
			*M*. *avium*	0.31 (0.3 *μ*M)	
Rufomycins 61			*M*. *tuberculosis* H37Rv	0.13 (0.13 *μ*M)	
			*M*. *abscessus*	0.6 (0.59 *μ*M)	
			*M*. *avium*	4.41 (4.3 *μ*M)	

Lunaemycin A	*S. lunaelactis* MM109T	Cave moonmilk deposits	*B*. *subtilis* ATCC 6633	0.12	[73]
			*S*. *pyogenes* ATCC 12344	0.12	
			*S*. *aureus* ATCC 25923	0.12	
			*S*. *aureus* ATCC 43300	0.12	
			*S*. *epidermidis* SI-1266	0.12	
			*S*. *haemolyticus* SI-6/2011	0.12	
			*Staphylococcus warneri* SI-5/2011	0.12	
			*E*. *faecalis* ATCC 29212	0.12	
			*E. faecalis* SI-759	0.25	
			*E*. *faecium* SI-1831	0.25	

Neo-actinomycin A	*Streptomyces* sp. IMB094	Marine sediment	*S. aureus* ATCC 29213 (MSSA)	16	[74]
			*S. aureus* 15 (MSSA)	32	
			*S. aureus* 13-17 (MSSA)	32	
			*S. aureus* ATCC 33591 (MRSA)	16	
			*S. aureus* 13-18 (MRSA)	64	
			*S. epidermidis* ATCC 12228 (MSSE)	64	
			*methicillin-sensitive S. epidermidis* 13–1 (MSSE)	64	
			*methicillin-resistant S. epidermidis* 13–3 (MRSE)	32	
			*E. faecalis* ATCC 29212 (VSE)	16	
			*E. faecalis* 13-4 (VSE)	16	
			*E. faecalis* ATCC 51299 (VRE)	16	
			*E. faecalis* ATCC 51575 (VRE)	16	
			*E. faecium* ATCC 700221 (VRE)	32	
			*E. faecium* 13-7 (VSE)	32	
			*E. faecium* 12-1 (VRE)	32	
Neo-actinomycin B			*S. aureus* ATCC 29213 (MSSA)	> 128	
			*S. aureus* 15 (MSSA)	> 128	
			*S. aureus* 13-17 (MSSA)	> 128	
			*S. aureus* ATCC 33591 (MRSA)	> 128	
			*S. aureus* 13-18 (MRSA)	> 128	
			*S. epidermidis* ATCC 12228 (MSSE)	> 128	
			*S. epidermidis* 13–1 (MSSE)	> 128	
			*S. epidermidis* 13–3 (MRSE)	> 128	
			*E. faecalis* ATCC 29212 (VSE)	> 128	
			*E. faecalis* 13-4 (VSE)	> 128	
			*E. faecalis* ATCC 51299 (VRE)	> 128	
			*E. faecalis* ATCC 51575 (VRE)	> 128	
			*E. faecium* ATCC 700221 (VRE)	> 128	
			*E. faecium* 13-7 (VSE)	> 128	
			*E. faecium* 12-1 (VRE)	> 128	

Actinomycin L_1_	*Streptomyces* sp. MBT27	Soil	*S. aureus* MB5393 (MRSA)	4–8	[75]
			*S. aureus* ATCC 29213	2–4	
			*E. faecium* (VSE)	4–8	
			*E. faecium* VanB (VRE)	4–8	
			*S. epidermidis*	4–8	
Actinomycin L_2_			*S. aureus* MB5393 (MRSA)	8–16	
			*E. faecium* (VSE)	8–16	
			*E. faecium* VanB (VRE)	8–16	

Svetamycins A and F	*Streptomyces* sp. DSM14386	Unknown	*S. aureus* ATCC 33582 (MRSA)	64	[76]
Svetamycins C and G			*S. aureus* ATCC 33582 (MRSA)	16	
Svetamycin E			*S. aureus* ATCC 33582 (MRSA)	> 64	

NW-G12	*S. alboflavus* 313	Soil	*B. cereus*	3.13	[77]
			*B. subtilis*	6.25	
			*S. aureus*	6.25	

Oleamycin A	*Streptomyces* sp. Lv20-58	Plant rhizosphere	*S. aureus*	0.23	[78]
			*M. luteus*	0.03	

Rufomycins NBZ1	*S*. *atratus* MJM3502	Unknown	*M*. *tuberculosis*	0.26 (0.25 *μ*M)	[79]
			*M*. *abscessus* ATCC 19977	—	
Rufomycin NBZ2			*M*. *tuberculosis*	0.45 (0.44 *μ*M)	
			*M*. *abscessus* ATCC 19977	—	
Rufomycin NBZ3			*M*. *tuberculosis*	0.87 (0.84 *μ*M)	
			*M*. *abscessus* ATCC 19977	9.67 (9.3 *μ*M)	
Rufomycin NBZ4			*M*. *tuberculosis*	> 9.67 (> 10 *μ*M)	
			*M*. *abscessus* ATCC 19977	> 9.67 (> 10 *μ*M)	
Rufomycin NBZ5			*M*. *tuberculosis*	0.119 (0.11 *μ*M)	
			*M*. *abscessus* ATCC 19977	0.39 (2.2 *μ*M)	
Rufomycin NBZ6			*M*. *tuberculosis*	0.57 (0.57 *μ*M)	
			*M*. *abscessus* ATCC 19977	> 10.14 (> 10 *μ*M)	
Rufomycin NBZ7			*M*. *tuberculosis*	0.10 (0.10 *μ*M)	
			*M*. *abscessus* ATCC 19977	0.57 (0.54 *μ*M)	
Rufomycins NBZ8			*M*. *tuberculosis*	0.031 (0.030 *μ*M)	
			*M*. *abscessus* ATCC 19977	0.61 (0.58 *μ*M)	

Marformycins A and C	*S. drozdowiczii* SCSIO 10141	Marine sediment	*M. luteus*	0.24	[80]
Marformycins B and E				4	
Marformycins D				0.063	

Albopeptide 6	*S. albofaciens* NCIMB 10975	Soil	*E. faecium* K60-39	0.78 (2.98 ± 0.07* μ*M)	[81]

Pentaminomycin C	*S*. *cacaoi* subsp. *cacaoi* NBRC 12748T	Unknown	*B*. *subtilis* subsp. *subtilis* NBRC 13719^T^ *S*. *aureus* subsp. *aureus* NBRC 100910^T^ *M*. *luteus* NBRC 3333^T^	16	[82]

NC-1	*Streptomyces* sp. FXJ1.172	Soil	*M. bovis* BCG	4.08 (44.4 *μ*M)	[83]

Quinomycin G	*Streptomyces* sp. LS298	Sponge	*S. epidermidis* ATCC 12228 (MSSE)	32	[84]
			*S. epidermidis* 12-6 (MSSE)	32	
			*S*. *epidermidis* 12-8 (MRSE)	32	
			*S. aureus* ATCC 29213 (MSSA)	32	
			*S. aureus* ATCC 33591 (MRSA)	32	
			*S. aureus* 15 (MSSA)	32	
			*S. aureus* 12-28 (MSSA)	32	
			*S. aureus* 12-33 (MRSA)	32	
			*E. faecium* ATCC 700221 (VRE)	32	
			*E. faecium* 12-1 (VRE)	32	
			*E. faecium* 12-3 (VSE)	64	
			*E. faecalis* ATCC 29212 (VSE)	16	
			*E. faecalis* ATCC 51299 (VRE)	16	
			*E. faecalis* 12-5 (VSE)	16	
			*E. faecalis* 09-9 (VRE)	16	

Skyllamycin D	*S. anulatus*	Plant endophyte	*B*. *subtilis* E168	8	[85]
			*S*. *aureus*	16	
Skyllamycin E			*B*. *subtilis* E168	32	
			*S*. *aureus*	64	

Desotamide B	*S. scopuliridis* SCSIO ZJ46	Marine sediment	*S. aureus* ATCC 29213	16	[86]
			*S. pneumoniae* NCTC 7466	12.5	
			*S*. *aureus* shhs-A1 (MRSA)	> 128	
			*S*. *epidermidis* shhs-E1 (MRSE)	32	
Desotamides C and D			*S. aureus* ATCC 29213	> 128	
			*S. pneumoniae* NCTC 7466	> 100	
			S. *aureus* shhs-A1 (MRSA)	> 128	
			*S*. *epidermidis* shhs-E1 (MRSE)	> 128	

**Table 5:** Lipopeptide products with anti-Gram-positive bacteria activity.

**Compound**	***Streptomyces* species/strain**	**Source**	**Tested Gram-positive bacteria**	**MIC (*μ*g/mL)**	**Reference**
Ambocidin A	*S. avermitilis* SUKA-17	Mutant	*B. subtilis* E168	< 0.031	[88]
			*S. aureus* ATCC 25923	0.25–0.5	
			*S. aureus* MRSA USA300	0.5–1	
			*E. faecium* (VRE)	0.25	
Ambocidin B			*Bacillus subtilis* E168	< 0.031	
			*S. aureus* ATCC 25923	4	
			*S. aureus* MRSA USA300	4	
			*E. faecium* (VRE)	2	

Gausemycin A	*Streptomyces* sp. INA-Ac-5812	Unknown	*S*. *aureus* ATCC 29213	0.5	[89]
			*S*. *aureus* ATCC 33592 (MRSA)	0.5	
			*S*. *epidermidis* ATCC 14990	0.25	
			*E*. *faecalis* ATCC 29212	> 64	
			*E*. *faecium* 3576 (VanR)	64	
			*Enterococcus gallinarum* 1308 (VanR)	> 64	
			*S*. *pneumonia* ATCC 49619	32	
			*S*. *pneumonia* ATCC 6305	> 32	
			*S. pyogenes* 7004	16	
			*S. pyogenes* P6	16	
			*S. pyogenes* P26	16	
			*S*. *agalactiae* S 17	> 32	
			*Streptococcus anginosus* Cp 16	> 32	
			*M*. *tuberculosis* H37Rv	> 40	
Gausemycin B			*S*. *aureus* ATCC 29213	1	
			*S*. *aureus* ATCC 33592 (MRSA)	1	
			*S*. *epidermidis* ATCC 14990	0.5	
			*E*. *faecalis* ATCC 29212	> 64	
			*E*. *faecium* 3576 (VRE)	> 64	
			*E. gallinarum* 1308 (VRE)	> 64	
			*S*. *pneumonia* ATCC 49619	> 32	
			*S*. *pneumonia* ATCC 6305	32	
			*S. pyogenes* 7004	32	
			*S. pyogenes* P6	32	
			*S. pyogenes* P26	32	
			*S*. *agalactiae* S 17	> 32	
			*S. anginosus* Cp 16	> 32	
			*M*. *tuberculosis* H37Rv	> 40	

Enamidonin B	*Streptomyces* sp. KCB14A132	Soil	*S. aureus* MRSA CCARM 3167	64	[90]
			*S. aureus* MRSA CCARM 3506	16	
			*S. aureus* QRSA CCARM 3505	32	
			*S. aureus* QRSA CCARM 3519	32	
			*S*. *aureus* RN 4220	32	
			*S*. *pneumoniae* KCTC 5412	> 128	
			*E. faecalis* KCTC 5191	64	
			*B. subtilis* KCTC 1021	16	
Enamidonin C			*S. aureus* MRSA CCARM 3167	32	
			*S. aureus* MRSA CCARM 3506	8	
			*S. aureus* QRSA CCARM 3505	16	
			*S. aureus* QRSA CCARM 3519	16	
			*S*. *aureus* RN 4220	> 128	
			*S*. *pneumoniae* KCTC 5412	> 128	
			*E. faecalis* KCTC 5191	8	
			*B. subtilis* KCTC 1021	16	

Aspartocin D	*S. canus* strain FIM-0916	Unknown	*B. subtilis*	0.125	[91]
			*S. aureus*	0.5	
Aspartocin E			*B. subtilis*	2	
			*S. aureus*	2	

A lipopeptide	*S. amritsarensis* sp. nov. MTCC 11845^T^	Soil	*B. subtilis* MTCC 619	10	[92]
			*M. smegmatis* MTCC 6	25	
			*S. epidermidis* MTCC 435	15	
			*S. aureus* MRSA	45	

Arylomycin A6	*Streptomyces* sp. HCCB10043	Unknown	*S. epidermidis* HCCB20256	1	[93]

Olenamidonin A	*S*. *olivaceus* SCSIO 1071	Marine sediment	*M*. *luteus* ML01	> 50	[94]
			*E. faecalis* CCARM 5172	3.12	
			*E*. *faecium* CCARM 5203	1.56	
Olenamidonin B			*M*. *luteus* ML01	> 50	
			*E. faecalis* CCARM 5172	1.56	
			*E*. *faecium* CCARM 5203	1.56	
Olenamidonin C			*M*. *luteus* ML01	> 50	
			*E. faecalis* CCARM 5172	6.25	
			*E*. *faecium* CCARM 5203	3.12	
Olenamidonin D			*M*. *luteus* ML01	> 50	
			*E. faecalis* CCARM 5172	3.12	
			*E*. *faecium* CCARM 5203	1.56	

Olikomycin A	*S. ghanaensis* ATCC 14672	Unknown	*S. aureus* 672234 (VRSA)	32	[95]
			*S. aureus* MRSA 2059512	32	
			*E. faecalis* 1894785	> 64	

**Table 6:** RiPP products with anti-Gram-positive bacteria activity.

**Compound**	***Streptomyces* species/strain**	**Source**	**Tested Gram-positive bacteria**	**MIC (*μ*g/mL)**	**Reference**
Globimycin	*S. globisporus subsp. globisporus* NRRL B-2709	Unknown	*B. subtilis*	0.5	[96]
			*S. aureus*	1	
			*M. luteus*	0.25	

Sviceucin	*S. sviceus* DSM 924	Unknown	*Bacillus megaterium*	1.3 (1.25 *μ*M)	[97]
			*E*. *faecalis* CIP 103015	10.4 (10 *μ*M)	
			*Lactobacillus brevis* F1.114	10.4 (10 *μ*M)	
			*Lactobacillus bulgaricus* 340	1.3 (1.25 *μ*M)	
			*Lactobacillus sakei* subsp. *sakei* DSM 20017	5.2 (5 *μ*M)	
			*Listeria ivanovii* subsp. *ivanovii* ATCC 19119	10.4 (10 *μ*M)	
			*L*. *monocytogenes*	> 10.4 (10 *μ*M)	
			*M. luteus* ATCC 9341	10.4 (10 *μ*M)	
			*S. aureus* subsp. *aureus* ATCC 6538	2.6 (2.5 *μ*M)	

Specialicin	*S. specialis* JCM GW41-1564^T^	Unknown	*M. luteus* NBRC 3333^T^	8	[98]

Cacaoidin	*S. cacaoi* CA-170360	Unknown	*Staphylococcus simulans 22*	0.25	[99]
			*S*. *aureus MB5393* (MRSA)	0.5	
			*S. aureus SG511*	2	
			*S. aureus SG511 DapR*	32	
			*M. tuberculosis H37Ra*	32	
			*B. subtilis 168*	8	
			*Clostridium difficile RyC 11872343*	8	
			*C. difficile RyC 11945271*	4	

Arcumycin	*Streptomyces* NRRL F-5639	Unknown	*B. subtilis* ATCC 6051	4	[100]
			*S*. *aureus* ATCC 25923	8	
			*M*. *luteus* 4698	8	
			*E*. *faecalis* ATCC 29212	> 32	

**Table 7:** Terpenoid products with anti-Gram-positive bacteria activity.

**Compound**	***Streptomyces* species/strain**	**Source**	**Tested Gram-positive bacteria**	**MIC (*μ*g/mL)**	**Reference**
Napyradiomycin 1	*Streptomyces* sp. SCSIO 10428	Marine sediment	*S. aureus* ATCC 29213	4	[101]
			*B. subtilis* SCSIO BS01	4	
			*B. thuringiensis* SCSIO BT01	8	
Napyradiomycin 2			*S. aureus* ATCC 29213	0.5	
			*B. subtilis* SCSIO BS01	1	
			*B. thuringiensis* SCSIO BT01	1	
Napyradiomycin 3			*S. aureus* ATCC 29213	> 128	
			*B. subtilis* SCSIO BS01	8	
			*B. thuringiensis* SCSIO BT01	16	

Merochlorin G	*Streptomyces* sp. CNH-189	Marine sediment	*B*. *subtilis* KCTC 1021	16	[102]
			*Kocuria rhizophila* KCTC 1915	32	
			*S*. *aureus* KCTC 1927	16	
Merochlorin H			*B*. *subtilis* KCTC 1021	64	
			*K. rhizophila* KCTC 1915	> 128	
			*S*. *aureus* KCTC 1927	> 128	
Merochlorin I			*B*. *subtilis* KCTC 1021	1	
			*K. rhizophila* KCTC 1915	2	
			*S*. *aureus* KCTC 1927	2	
Merochlorin J			*B*. *subtilis* KCTC 1021	> 128	
			*K. rhizophila* KCTC 1915	> 128	
			*S*. *aureus* KCTC 1927	> 128	

Chlorolabdan A	*S*. *griseorubens* 2210JJ-087	Marine sediment	*B*. *subtilis* KCTC 102	> 128	[103]
			*M*. *luteus* KCTC 1915	> 128	
			*S*. *aureus* KCTC 1927	> 128	
Chlorolabdan B			*B*. *subtilis* KCTC 102	4	
			*M*. *luteus* KCTC 1915	8	
			*S*. *aureus* KCTC 1927	8	
Chlorolabdan C			*B*. *subtilis* KCTC 102	32	
			*M*. *luteus* KCTC 1915	> 128	
			*S*. *aureus* KCTC 1927	> 128	
Epoxylabdans A and B			*B*. *subtilis* KCTC 102	> 128	
			*M*. *luteus* KCTC 1915	> 128	
			*S*. *aureus* KCTC 1927	> 128	

Napyradiomycin 1	*Streptomyces* sp. CA-271078	Marine	*S. aureus* MB5393 (MRSA)	> 96	[104]
			*M. tuberculosis* H37Ra	—	
Napyradiomycin 2			*S. aureus* MB5393 (MRSA)	48	
			*M. tuberculosis* H37Ra	12–24	
Napyradiomycin 3			*S. aureus* MB5393 (MRSA)	> 64	
			*M. tuberculosis* H37Ra	> 64	
Napyradiomycin 5			*S. aureus* MB5393 (MRSA)	> 96	
			*M. tuberculosis* H37Ra	24–48	

Napyradiomycin A	*Streptomyces* sp. CNQ-329	Marine sediment	*S. aureus* (MRSA)	16	[105]
Napyradiomycin B			*S. aureus* (MRSA)	64	
Napyradiomycins C-F			*S. aureus* (MRSA)	> 64	

Napyradiomycin A4	*S*. *kebangsaanensis* WS-68302	Soil	*S*. *aureus* ATCC 25923	25	[106]
			*Erysipelothrix rhusiopathiae*	50	
			WH13013 *Streptococcus suis* SC19	100	

**Table 8:** Polyketide–terpenoid products with anti-Gram-positive bacteria activity.

**Compound**	***Streptomyces* species/strain**	**Source**	**Tested Gram-positive bacteria**	**MIC (*μ*g/mL)**	**Reference**
Flaviogeranin B1	*Streptomyces* sp. B9173	Marine sediment	*S*. *aureus* ATCC 43300	14.6	[107]
			*M*. *smegmatis* MC2 255	12.4	
Flaviogeranin B			*S*. *aureus* ATCC 43300	28.1	
			*M*. *smegmatis* MC2 255	35.1	
Flaviogeranin D			*S*. *aureus* ATCC 43300	9.2	
			*M*. *smegmatis* MC2 255	5.2	

Furaquinocin K	*Streptomyces* sp. Je 1-369	Plant rhizosphere	*B*. *subtilis* DSM 10	> 64	[108]
			*S*. *aureus* Newman	> 64	
			*M*. *smegmatis* mc2155	> 64	
Furaquinocin L			*B*. *subtilis* DSM 10	64	
			*S*. *aureus* Newman	2	
			*M*. *smegmatis* mc2155	> 64	

**Table 9:** Alkaloid products with anti-Gram-positive bacteria activity.

**Compound**	***Streptomyces* species/strain**	**Source**	**Tested Gram-positive bacteria**	**MIC (*μ*g/mL)**	**Reference**
Streptopyrrole B	*S. zhaozhouensis* 208DD-064	Marine sediment	*B*. *subtilis* KCTC 1021	0.26 (0.8 *μ*M)	[109]
			*M*. *luteus* KCTC 1915	0.26 (0.8 *μ*M)	
			*S*. *aureus* KCTC 1927	0.26 (0.8 *μ*M)	
Streptopyrrole C			*B*. *subtilis* KCTC 1021	0.98 (2.9 *μ*M)	
			*M*. *luteus* KCTC 1915	0.23 (0.7 *μ*M)	
			*S*. *aureus* KCTC 1927	0.23 (0.7 *μ*M)	

Dionemycin	*Streptomyces* sp. SCSIO 11791	Marine sediment	*M*. *luteus* ML01 Ju1	0.5	[110]
			*S*. *aureus* ATCC 29213	1	
			*S. aureus* MRSA 991	2	
			*S. aureus* MRSA 1862	2	
			*S. aureus* MRSA 669A	2	
			*S. aureus* MRSA A2	2	
			*S. aureus* MRSA GDQ6P012P	2	
			*S. aureus* MRSA GDE4P037P	2	
6-OMe-7⁣′,7⁣^″^-dichorochromopyrrolic acid			*M*. *luteus* ML01 Ju1	16	
			*S*. *aureus* ATCC 29213	3	
			*S. aureus* MRSA 991	64	
			*S. aureus* MRSA 1862	32	
			*S. aureus* MRSA 669A	32	
			*S. aureus* MRSA A2	32	
			*S. aureus* MRSA GDQ6P012P	128	
			*S. aureus* MRSA GDE4P037P	64	

Strepchazolin A	*S*. *chartreusis* NA02069	Marine sediment	*B*. *subtilis*	15.7 (64 *μ*M)	[111]
Strepchazolin B				31.4 (> 128 *μ*M)	

**Table 10:** Phenazine products with anti-Gram-positive bacteria activity.

**Compound**	***Streptomyces* species/strain**	**Source**	**Tested Gram-positive bacteria**	**MIC (*μ*g/mL)**	**Reference**
Phenazine SA	*Streptomyces* sp. NA04227	Earwig endophyte	*M. luteus*	> 37.7 (> 128 *μ*M)	[112]
Phenazine SB				37.7 (128 *μ*M)	
Phenazine SC				1.48 (4 *μ*M)	

Hydroxy-7-oxolavanducyanin	*Streptomyces* sp. CPCC 203577	Soil	*S. epidermidis* ATCC 12228 (MSSE)	0.06	[113]
			*S. aureus* ATCC 29213 (MSSA)	8	
			*S. aureus* ATCC 33591 (MRSA)	8	
			*E*. *faecalis* ATCC 29212	> 16	

Diastaphenazine	*S. diastaticus* subsp. *ardesiacus*	Plant endophyte	*S. aureus* ATCC 25923	64	[114]

Baraphenazine A	*Streptomyces sp.* PU-10A	Soil	*S*. *aureus* ATCC 6538	> 49.2	[115]
			*M*. *luteus* NRRL B-287	> 49.2	
			*B*. *subtilis* ATCC 6633	> 49.2	
			*M*. *aurum* ATCC 23366	> 49.2	
Baraphenazines B and C			*S*. *aureus* ATCC 6538	> 52	
			*M. luteus* NRRL B-287	> 52	
			*B*. *subtilis* ATCC 6633	> 52	
			*M*. *aurum* ATCC 23366	> 52	
Baraphenazine D			*S*. *aureus* ATCC 6538	> 52.3	
			*M. luteus* NRRL B-287	> 52.3	
			*B*. *subtilis* ATCC 6633	> 52.3	
			*M*. *aurum* ATCC 23366	> 52.3	
Baraphenazine E			*S*. *aureus* ATCC 6538	1.7	
			*M. luteus* NRRL B-287	3.3	
			*B*. *subtilis* ATCC 6633	3.3	
			*M*. *aurum* ATCC 23366	13.1	
Baraphenazines F and G			*S*. *aureus* ATCC 6538	> 56.2	
			*M. luteus* NRRL B-287	> 56.2	
			*B*. *subtilis* ATCC 6633	> 56.2	
			*M*. *aurum* ATCC 23366	> 56.2	

**Table 11:** Nucleoside antibiotic products with anti-Gram-positive bacteria activity.

**Compound**	***Streptomyces* species/strain**	**Source**	**Tested Gram-positive bacteria**	**MIC (*μ*g/mL)**	**Reference**
Mavintramycin A	*Streptomyces* sp. OPMA40551	Unknown	*M*. *avium* JCM15430	0.78	[116]
			*M*. *intracellulare* JCM6384	0.39	
			*M*. *smegmatis* MC^2^ 155	3.12	
			*M*. *bovis* BCG	1.56	
Mavintramycin B			*M*. *avium* JCM15430	6.25	
			*M*. *intracellulare* JCM6384	3.12	
			*M*. *smegmatis* MC^2^ 155	12.5	
			*M*. *bovis* BCG	6.25	
Mavintramycin C			*M*. *avium* JCM15430	> 50	
			*M*. *intracellulare* JCM6384	25	
			*M*. *smegmatis* MC^2^ 155	> 50	
			*M*. *bovis* BCG	6.25	
Mavintramycin D			*M*. *avium* JCM15430	25	
			*M*. *intracellulare* JCM6384	6.25	
			*M*. *smegmatis* MC^2^ 155	25	
			*M*. *bovis* BCG	6.25	
Mavintramycin E			*M*. *avium* JCM15430	12.5	
			*M*. *intracellulare* JCM6384	12.5	
			*M*. *smegmatis* MC^2^ 155	50	
			*M*. *bovis* BCG	6.25	
Mavintramycin F			*M*. *avium* JCM15430	3.12	
			*M*. *intracellulare* JCM6384	0.78	
			*M*. *smegmatis* MC^2^ 155	3.12	
			*M*. *bovis* BCG	1.56	
Mavintramycin G			*M*. *avium* JCM15430	3.12	
			*M*. *intracellulare* JCM6384	0.78	
			*M*. *smegmatis* MC^2^ 155	12.5	
			*M*. *bovis* BCG	0.39	

Liposidomycin congener 1	*Streptomyces* sp. TMPU-20A065	Soil	*M*. *avium*	64	[117]
			*M*. *intracellulare*	4	
			*M*. *bovis* BCG	> 64	
			*M*. *smegmatis*	> 64	
			*B*. *subtilis*	> 64	
			*S*. *aureus*	> 64	
Liposidomycin congener 2			*M*. *avium*	16	
			*M*. *intracellulare*	8	
			*M*. *bovis* BCG	> 64	
			*M*. *smegmatis*	> 64	
			*B*. *subtilis*	> 64	
			*S*. *aureus*	> 64	
Liposidomycin congener 4			*M*. *avium*	32	
			*M*. *intracellulare*	4	
			*M*. *bovis* BCG	> 64	
			*M*. *smegmatis*	> 64	
			*B*. *subtilis*	> 64	
			*S*. *aureus*	> 64	

Streptcytosine A	*Streptomyces* sp. TPU1236A	Marine	*M. smegmatis* NBRC 3207	32	[118]

**Table 12:** Other natural products with anti-Gram-positive bacteria activity.

**Compound**	***Streptomyces* species/strain**	**Source**	**Tested Gram-positive bacteria**	**MIC (*μ*g/mL)**	**Reference**
Pyrimidomycin	*Streptomyces* sp. PSAA01	Soil	*S. aureus* MTCC 96	12	[119]
			*S. pyogenes* MTCC 1928	12	
			*B. cereus* MTCC 1272	12	
			*S. aureus* (MRSA)	12	
			*M. smegmatis* MC^2^ 155	12	
			*B. subtilis* MTCC 441	24	

Mersaquinone	*Streptomyces* sp. EG1	Marine sediment	*S. aureus* MRSA TCH1516	3.36	[120]

Youssoufenes A2 and A3	*S*. *youssoufiensis* OUC6819	*Δ*dtlA Mutant	*E*. *faecalis* CCARM 5172	12.5 (22.2 *μ*M)	[121]

Aryl-C-glycoside 1	*Streptomyces* sp. OUCMDZ-945	Soil	*S. aureus* ATCC 6538	8	[122]
			*S. aureus* MRSA ATCC 43300	4	
Aryl-C-glycoside 2			*S. aureus* ATCC 6538	32	
			*S. aureus* MRSA ATCC 43300	64	
Aryl-C-glycoside 5			*S. aureus* ATCC 6538	> 64	
			*S. aureus* MRSA ATCC 43300	16	
Aryl-C-glycoside 6			*S. aureus* ATCC 6538	64	
			*S. aureus* MRSA ATCC 43300	16	
Aryl-C-glycoside 7			*S. aureus* ATCC 6538	16	
			*S. aureus* MRSA ATCC 43300	8	

Fradiamine A	*S. fradiae* MM456M-mF7	Marine sediment	*Clostridium bifermentans*	> 128	[123]
			*C*. *butyricum*	64	
			*C. difficile*	32	
			*C. indolis*	> 128	
			*C*. *innocuum*	> 128	
			*C*. *limosum*	> 128	
			*C*. *perfringens*	> 128	
			*C*. *ramosum*	> 128	
Fradiamine B			*C. bifermentans*	> 128	
			*C*. *butyricum*	128	
			*C. difficile*	8	
			*C. indolis*	16	
			*C*. *innocuum*	32	
			*C*. *limosum*	128	
			*C*. *perfringens*	> 128	
			*C*. *ramosum*	64	

Aurachin SS	*Streptomyces* sp. NA04227	Earwig endophyte	*S. aureus*	20.8 (64 *μ*M)	[124]
			*S. aureus* (MRSA)	> 41.6 (> 128 *μ*M)	
			*S. pyogenes*	10.4 (32 *μ*M)	
			*B. subtilis*	20.8 (64 *μ*M)	
			*M. luteus*	10.4 (32 *μ*M)	

Catechol siderophore	*S. varsoviensis* CGMCC 4.1431	Unknown	*L. monocytogenes* AB97021	25	[125]

7-Prenylisatin	*Streptomyces* sp. MBT28	Unknown	*B. subtilis*	25	[126]

1,3-Benzodioxole	*Streptomyces* sp. G261	Marine sediment	*E. faecalis* ATCC13124	128	[127]
			*S. aureus* ATCC25923	256	

16-Deethylindanomycin	*S*. *antibioticus* PTZ0016	Marine sediment	*S*. *aureus* ATCC 6538	4	[128]
Iso-16-deethylindanomycin				6	
16-Deethylindanomycin methyl ester				6	
Iso-16-deethylindanomycin methyl ester				8	

## Data Availability

Data sharing is not applicable to this article as no new data were created or analyzed in this study.

## Supporting Information

Supporting Information Additional supporting information can be found online in the Supporting Information section. Additional supporting information is provided in the Supporting Information section, including the PRISMA 2020 checklist that guided the reporting of this review.
